# Supplementary material for: Target-Specific Precision of CRISPR-Mediated Genome Editing
Source: Mol Cell. 2019 Feb 21;73(4):699–713.e6. doi: 10.1016/j.molcel.2018.11.031 (PMC6395888; doi:10.1016/j.molcel.2018.11.031)
Supplement: Document S1. Figures S1–S7 and Table S5 [file mmc1.pdf]

Molecular Cell, Volume *73*

Supplemental Information

Target-Specific Precision

of CRISPR-Mediated Genome Editing

Anob M. Chakrabarti, Tristan Henser-Brownhill, Josep Monserrat, Anna R. Poetsch, Nicholas M. Luscombe, and Paola Scaffidi

Figure S1. Related to Figure 1

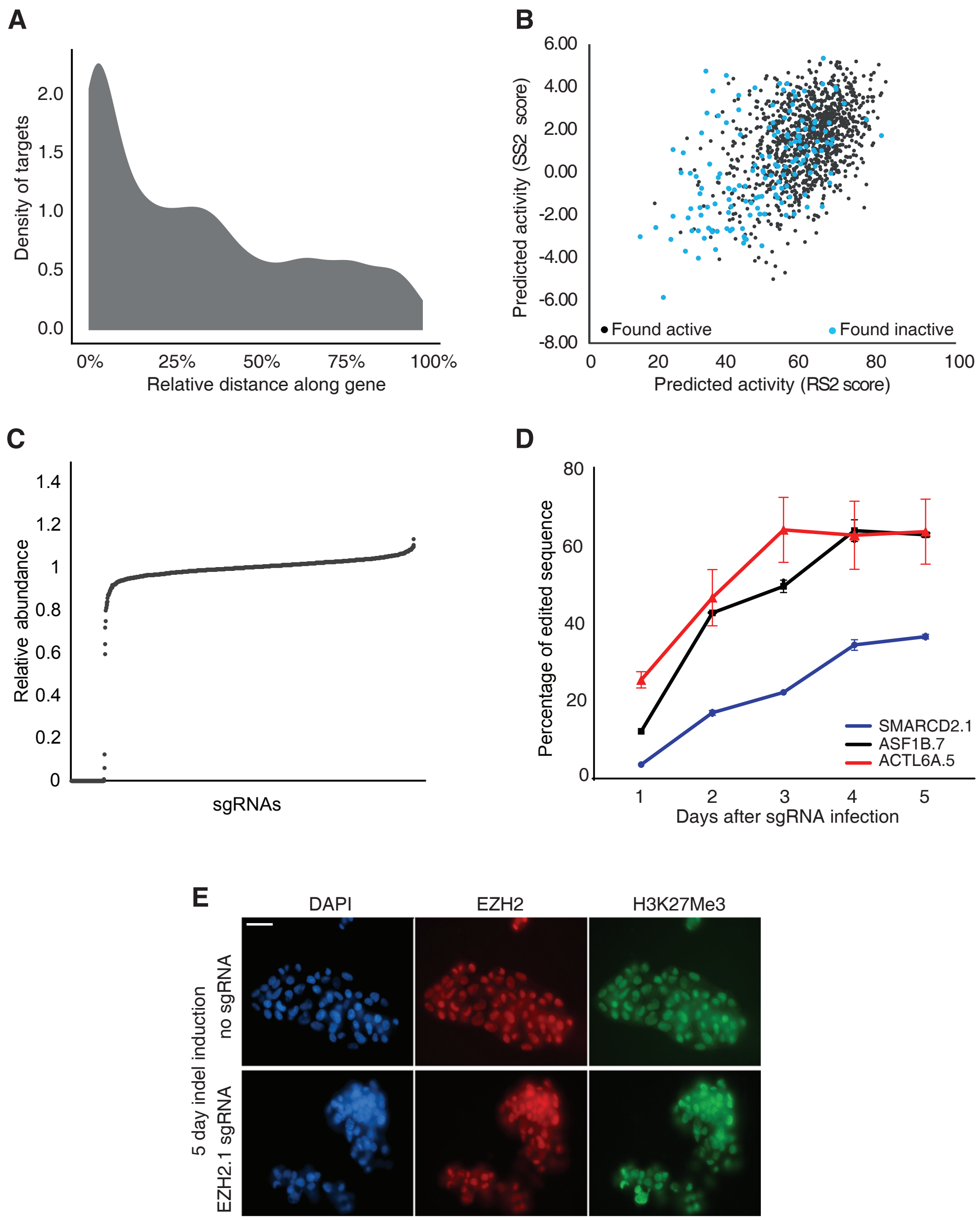

Figure S1. Characteristics of the sgRNA pools. Related to Figure 1.

A. Distribution of cleavage site locations of all target sites along the gene body.

B. Correlation between the indicated scores of predicted sgRNA activity for the used guides obtained from two distinct algorithms (Chari et al., 2017; Doench et al., 2016). sgRNAs that induced detectable indels are shown in black, while inactive sgRNAs are in blue. Note that several sgRNAs with high predicted activity were found to be inactive. Only sgRNAs with counts > 0 in C are shown.

C. Relative abundance of each sgRNA in the 450 pools. Values represent sequencing reads normalized to the median value of the three pools combined. With the exception of a few undetectable sgRNAs, all guides are homogeneously represented in the pools.

D. Time course of CRISPR-mediated editing at the indicated sites, after infection of individual sgRNAs. Values are mean ± standard deviation from two technical replicates. The percentage of edited sequence was estimated by TIDE analysis (Brinkman et al., 2014).

E. Immunofluorescence microscopy of the indicated samples using anti-EZH2 (red) and anti-H3K27me3 (green) antibodies, showing no detectable reduction in EZH2 or histone modification levels 5 days after indel induction. Nuclei are counterstained with DAPI (blue). Scale Bar 20 µm. EZH2.1 sgRNA had been individually transduced in Cas9-expressing cells using the same conditions used for the pooled sgRNAs, serving as a reporter of indel formation efficiency in the large-scale experiment. TIDE analysis of genomic DNA showed an editing efficiency of 37.6% at the EZH2.1 site.

Figure S2. Related to Figure 1

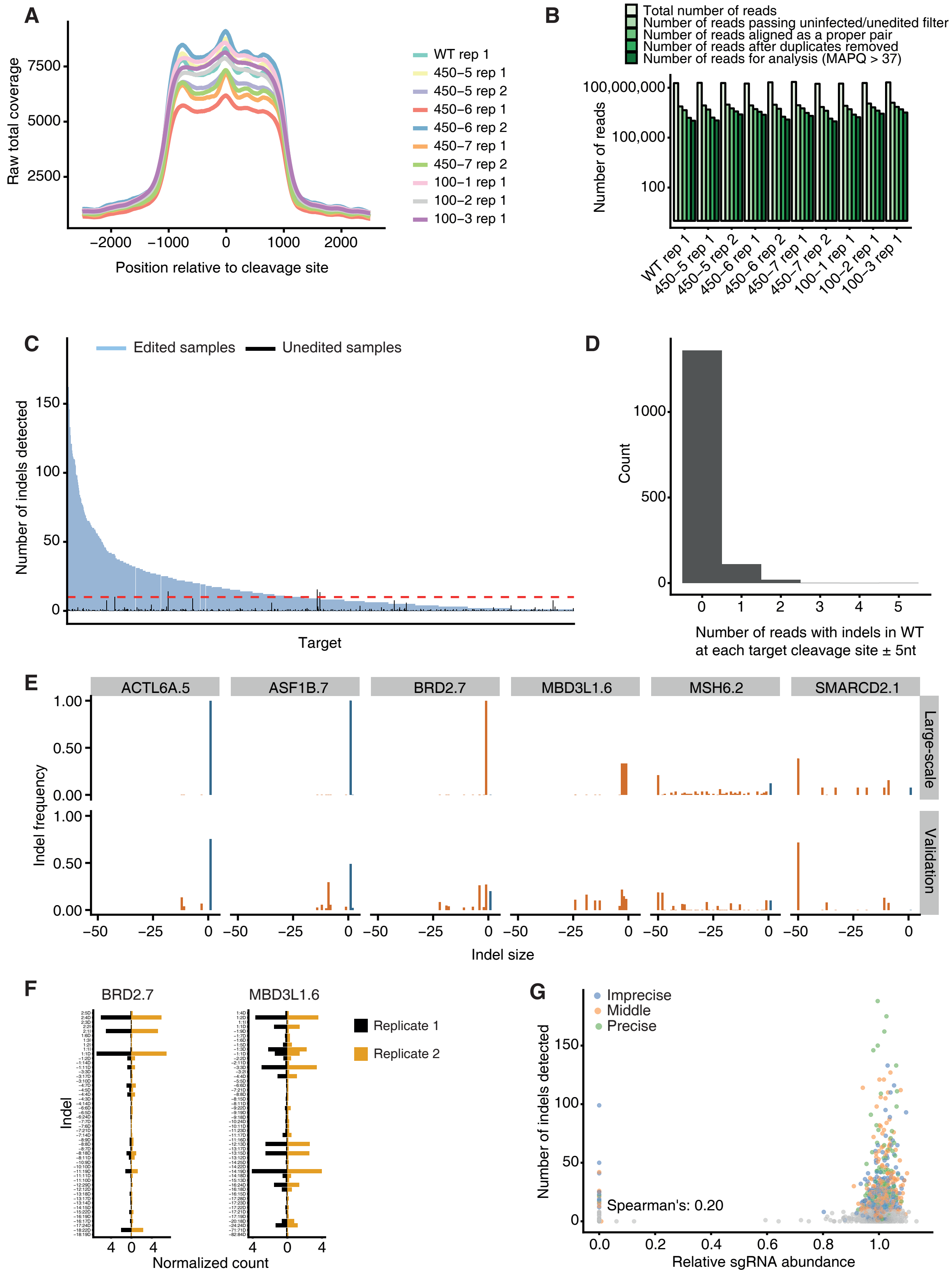

Figure S2. Indel detection metrics and validation of indel profiles. Related to Figure 1.

A. Raw total read coverage for each experiment over the region around the cleavage site that was selectively isolated by target capture.

B. Alignment metrics for the large-scale experiments. For each experiment, the number of reads at each stage of processing is shown. The filtering strategy used to detect indels robustly is described in the Methods section.

C. Number of total indels detected at each target site when using the 450 sgRNA pools (summed across both biological replicates). The number of indels detected in unedited control samples (see methods) is shown in black. The dashed line is at 10, reflecting the threshold that was set for the downstream analysis.

D. Number of indels detected at target sites in the wild-type experiment without Cas9 induction; data from one biological replicate.

E. Comparison of indels detected at 6 target sites in the large-scale experiment and the validation experiment.

F. Indel profiles for two biological replicates at the indicated target sites probed by high-coverage sequencing in validation experiments. Indel nomenclature: is [start coordinate relative to cleavage site]:[size][insertion or deletion]. Counts are normalized to the total library size for each experiment.

G. Relationship between the number of total indels detected at each target site and the abundance of the associated sgRNA in the pools. Note that some sgRNAs that were undetectable in the pools by next-generation sequencing induced indels at their target sites. Presence of the undetected, indel-inducing sgRNAs was confirmed by Sanger sequencing of the individual guides in the original arrayed library. These “undetectable” sgRNAs are included in C. sgRNAs are color-coded based on the groups described in Fig. 3A. Grey targets are those excluded from the downstream analysis as they induced less than 10 indels.

Figure S3. Related to Figures 2 & 3

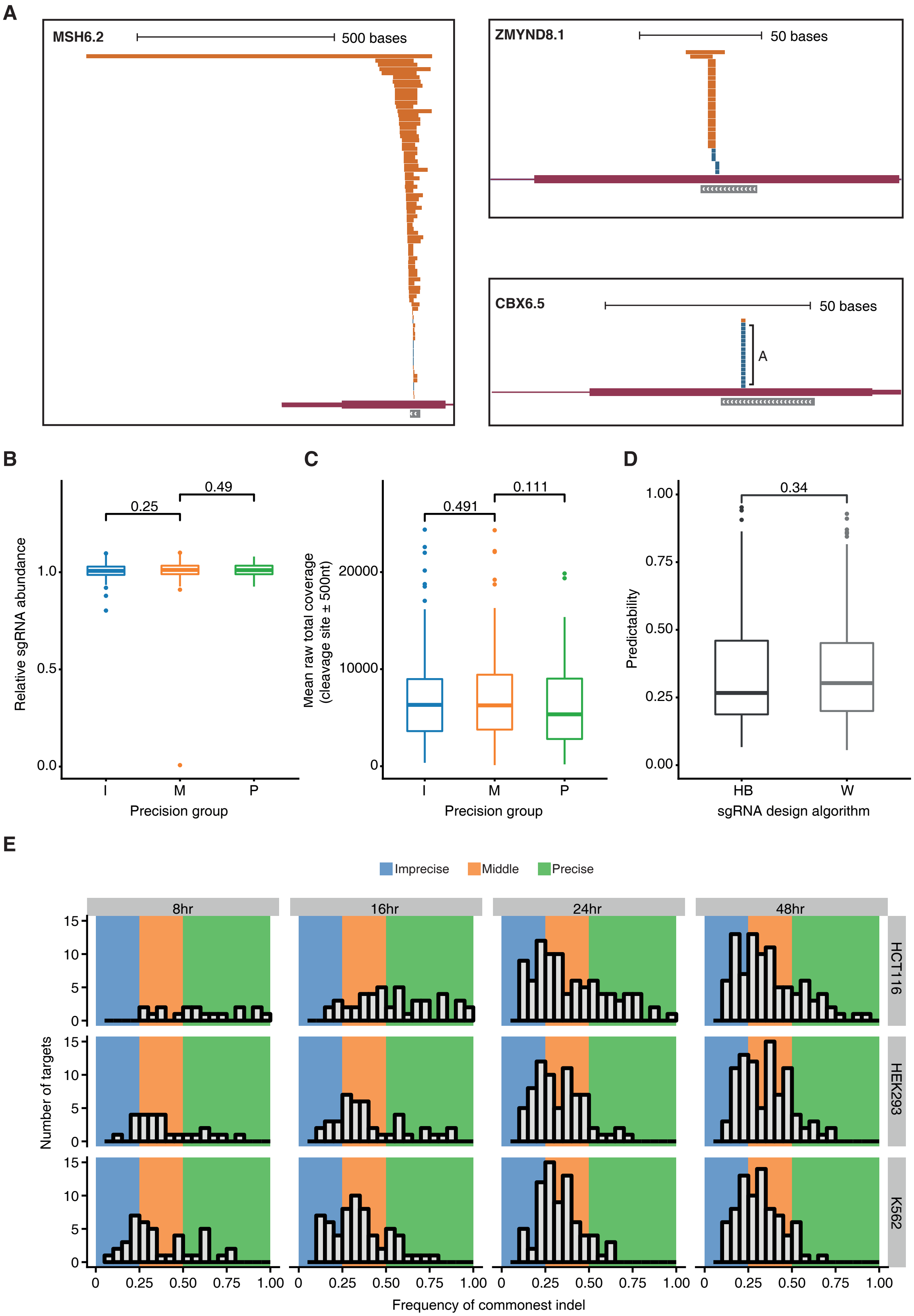

Figure S3. Editing precision groups of targets. Related to Figures 2 & 3.

A. Example sites showing a wide range of distinct indels (MSH6.2) or strong preference for a specific deletion (ZMYND8.1) or insertion (CBX6.5). Deletions are shown in orange, insertions in blue, with the gene body colored in plum and the sgRNA binding position in grey. Chevrons indicate strand.

B-C. Relationship between sgRNA abundance (B) and raw total read coverage (C) and assignment of precision group to target sites. Statistical testing: Kruskal-Wallis test followed by Dunn's test for multiple comparisons with Benjamini-Hochberg correction for multiple testing.

D. Relationship between the sgRNA design algorithm and assignment of precision group to target sites. HB and W indicate Henser-Brownhill design (purely based on sgRNA specificity) and Wang design (optimized for activity), respectively (Henser-Brownhill et al., 2017). Statistical testing: Kruskal-Wallis test.

E. Distribution of commonest indel frequencies at target sites from the van Overbeek dataset (van Overbeek et al., 2016) at the indicated times after sgRNA nucleofection. The background indicates three groups of sites as defined based on their editing precision. Note that a stable distribution is observed by 24h. Targets with an editing efficiency of < 10% at a time-point have been filtered out. The 4h time point has been omitted as no sites passed the threshold for HEK293.

Figure S4. Related to Figures 5

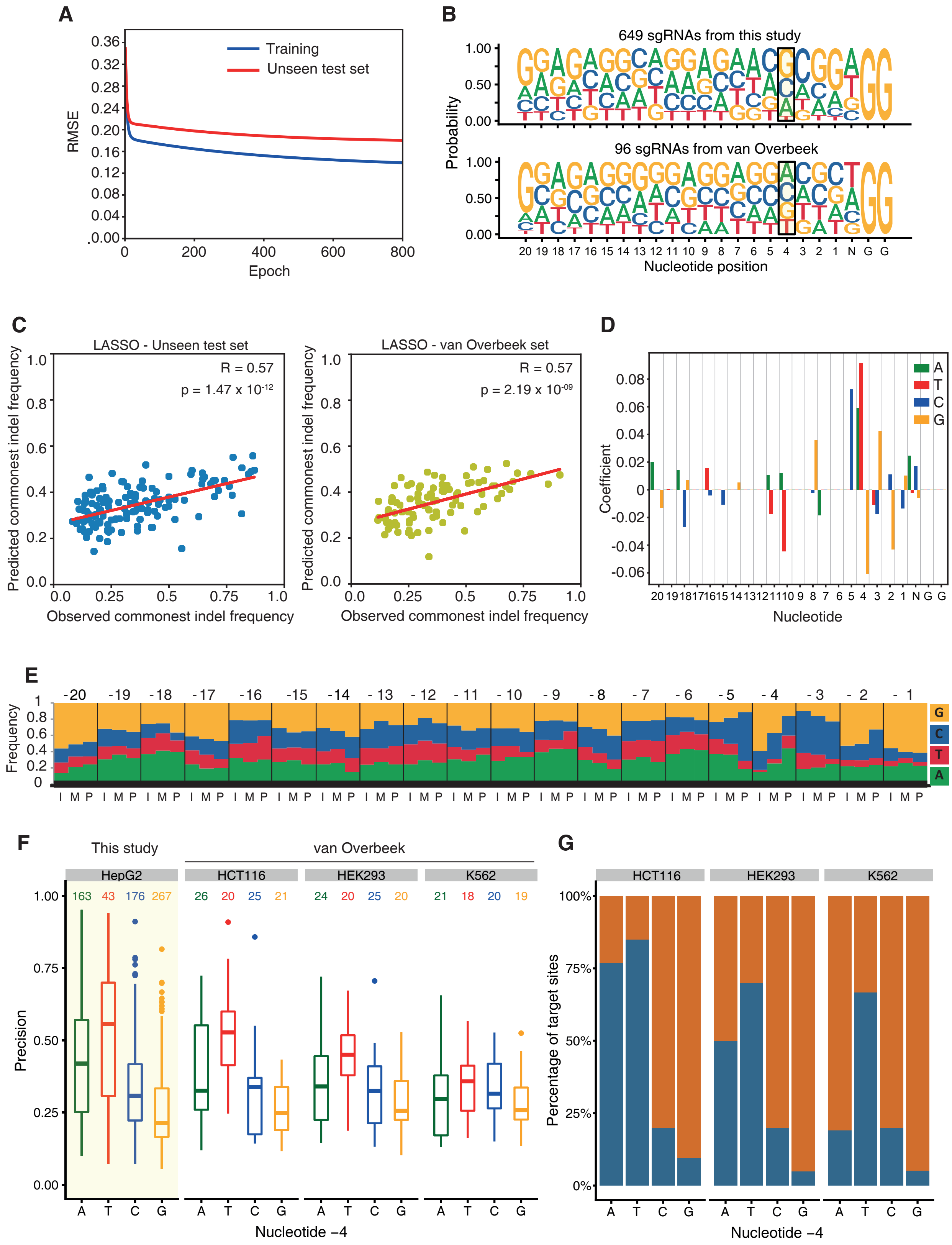

Figure S4. Robust predictability of editing outcome based on protospacer nucleotide composition. Related to Figure 5.

A. Final model training for the artificial neural network, with corresponding test error. Epoch indicates the number of iterations the network was presented with the training dataset.

B. Sequence logo showing the distribution of nucleotides at each position of the sgRNAs used here (above) and used by van Overbeek *et al.* (below).

C. Correlation between the observed precision at a given target site and that estimated by the LASSO model, using our test set (left) and the van Overbeek set (right). The best model selected after 10-fold cross-validation is shown (see Methods). R: correlation coefficient. Statistical significance testing: Wald χ^2 test.

D. Contribution of each nucleotide of the protospacer to editing precision as estimated by the LASSO model. Positive and negative values indicate that the nucleotide favor or disfavor precision, respectively.

E. Frequencies of each nucleotide in the protospacer sequence for each precision group. I: imprecise, M: middle, P: precise.

F. Precision of targets with the indicated nucleotide at position -4 in the sites targeted here (HepG2, shaded in yellow) compared with that calculated for the 96 van Overbeek *et al.* target sites in 3 different cell lines at the 48h time point.

G. Percentage of target sites with a preference (commonest indel) for insertions or deletions based on the nucleotide at position -4 calculated for the 96 van Overbeek *et al.* target sites in 3 different cell lines.

Figure S5. Related to Figures 6 & 7

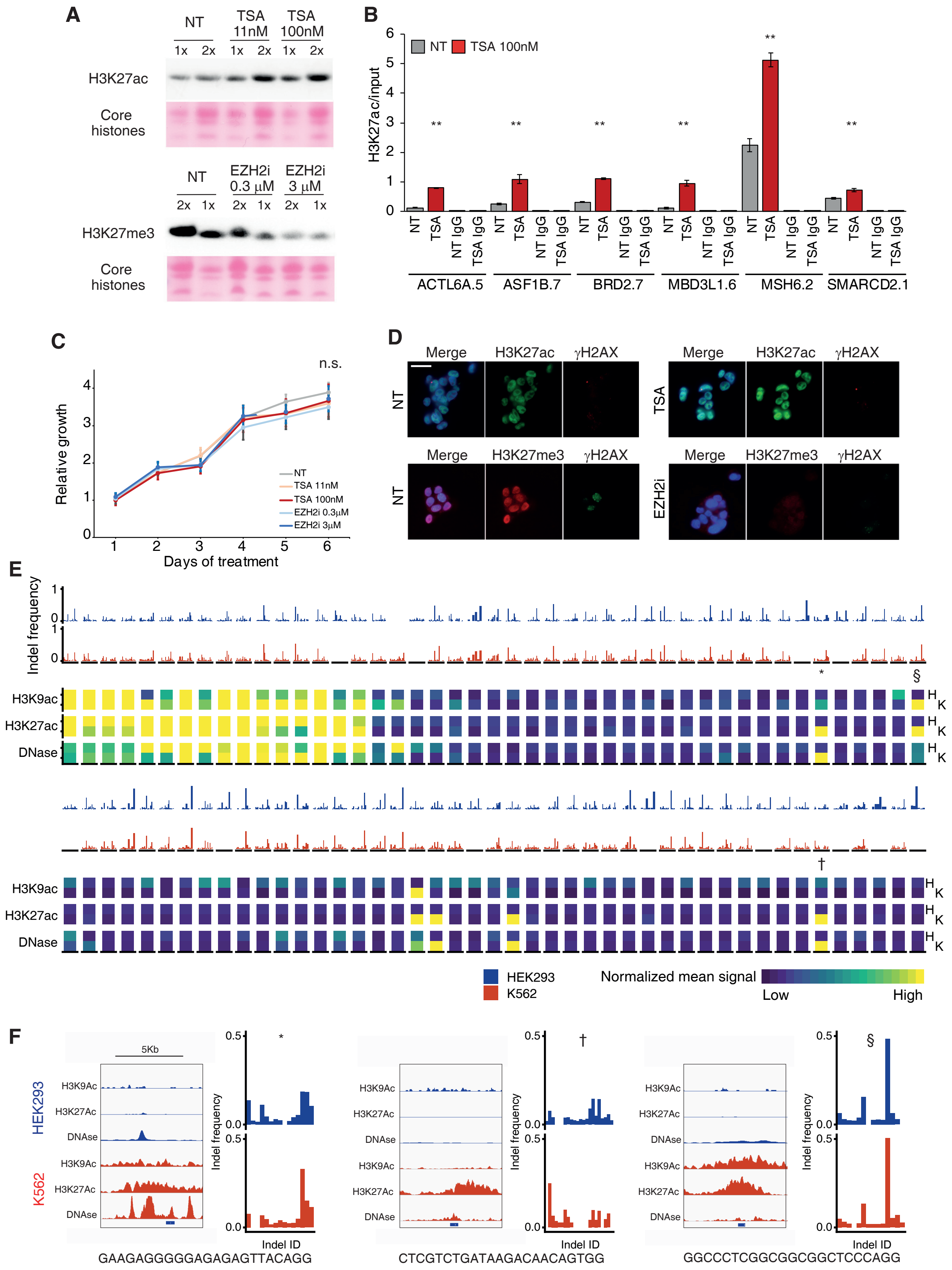

Figure S5. Chromatin modulation by TSA and EZH2i treatment. Related to Figures 6 & 7.

A. Western blot analysis of HepG2 cells untreated (NT) or treated with the indicated compounds. A dose-dependent increase in H3K27ac is observed in response to TSA (above), while EZH2i induces a dose-dependent reduction in H3K27me3 (below). Ponceau S staining of the core histones is shown as loading control. Two different amounts of protein lysate (1x and 2x) are loaded for each condition to allow a more quantitative assessment of the differences.

B. ChIP-qPCR showing increase of H3K27ac upon TSA treatment at the indicated sites. Values are mean ± standard deviation from two independent experiments. Values of pulled-down chromatin are normalized to 10% input chromatin. Statistical testing: Mann Whitney test (p value of < 0.01 shown as two asterisks). Signal for H3K27ac was enriched compared to IgGs for all sites in untreated (NT) cells. Similar experiments were performed upon EZH2i treatment, but due to intrinsically low levels of this mark in HepG2 cells we were unable to detect H3K27me3 at the sites. Even positive control genes (e.g. WT1 or HOXB9 promoter) showed minimal enrichment over the IgG control, while they showed strong enrichment in human fibroblasts used as a control cell line (not shown).

C. Proliferation curves of HepG2 cells treated with the indicated compounds. Values are mean ± standard deviation of three biological replicates. No significant (n.s.) difference in cell growth is observed. Statistical testing: Mann Whitney test on the last time point.

D. Immunofluorescence microscopy of the indicated samples using the indicated antibodies, showing no detectable change in endogenous DNA damage, as assessed by staining for γH2A.X. Nuclei are counterstained with DAPI (blue). Scale Bar 10 µm.

E. Above, indel profiles at 96 target sites from van Overbeek *et al.* Below, normalized ChIP-seq signal for H3K9ac and H3K27ac and DNase-seq signal in a 500-nucleotide window centred on the cleavage site. Data presented from HEK293 (H) and K562 (K) cells (Cistrome DB 43073, 45020, 45021, 45406, 55731, 58997 and GEO GSM1635901 - 6).

F. Examples of two imprecise (left and middle) and one precise (right) sites showing highly different chromatin states in the indicated cell lines. For each target, tracks of the indicated histone marks and DNAse hypersensitivity sites (left) and the indel profiles (right) in the two cell lines are shown. The location of target sites is shown in blue below the tracks.

Figure S6. Related to Figure 6

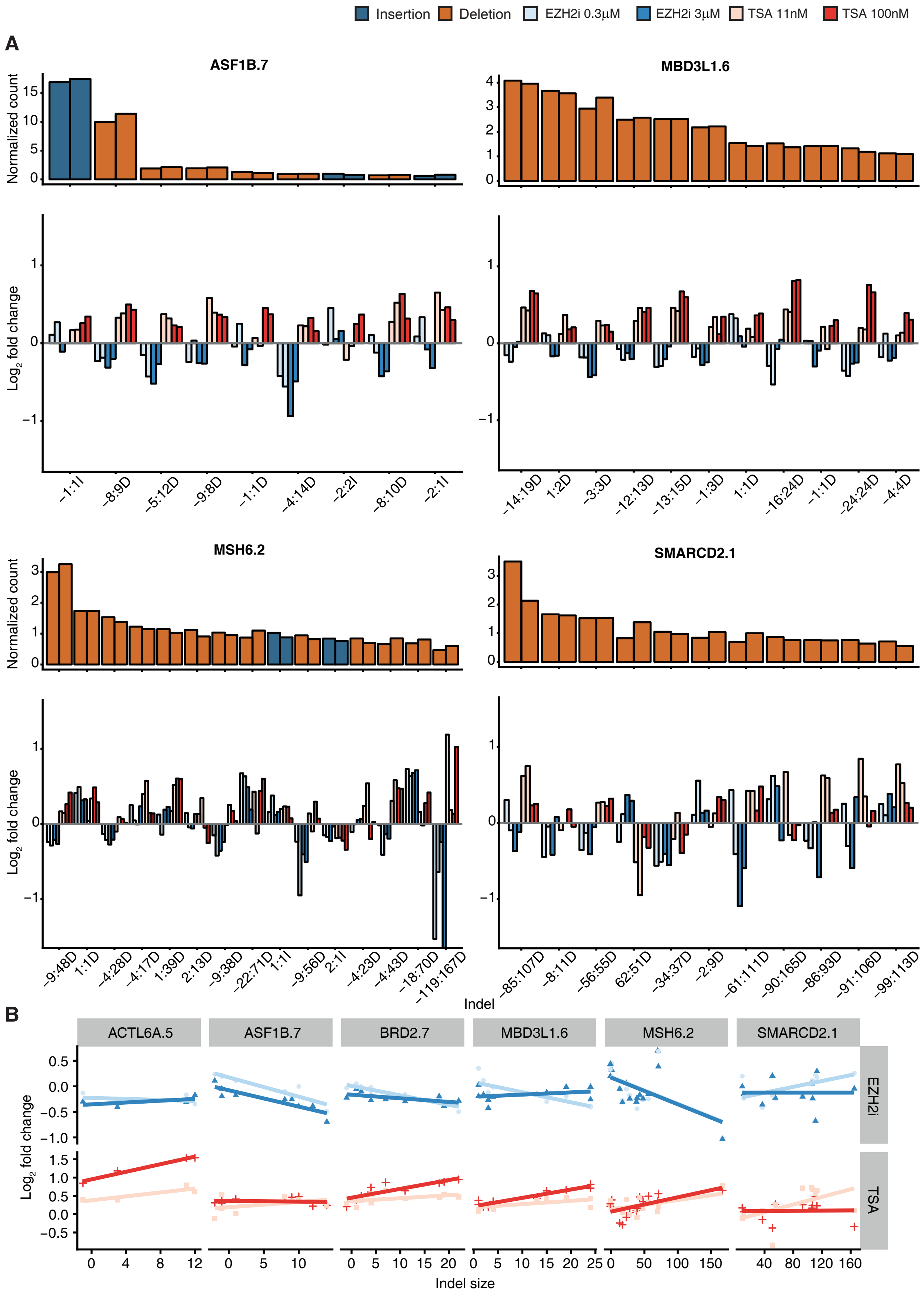

Figure S6. Chromatin modulation affects RGN activity. Related to Figure 6.

A. As for Figure 6D, the remaining 4 target sites show that chromatin modulation affects both insertions and deletions. Above is shown the count of each indel, normalized by the effective library size at each site for each replicate. Only indels with a normalized count of at least 1 in any condition are included.

B. Log_2-fold change for each size of indel for each target site and for each condition.

Figure S7. Related to Figure 7

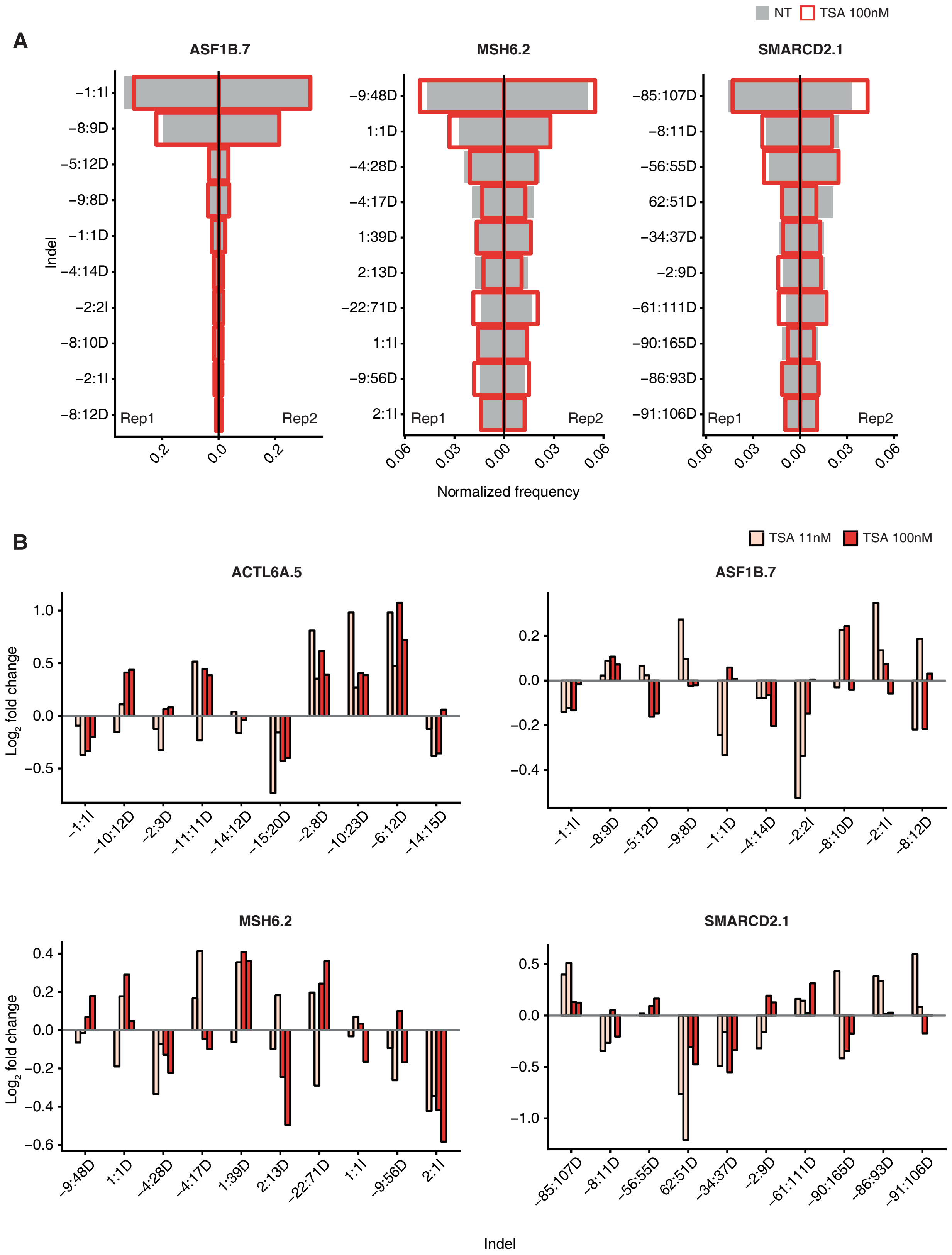

Figure S7. Chromatin modulation affects indel profiles. Related to Figure 7.

A. As for Fig. 7A, the remaining 3 targets show that chromatin modulation has a differential effect on distinct indels. Count for each indel, normalized by the total number of indels detected at that target site in that condition for each replicate. The frequency of the indicated indels in the untreated condition (grey bars) and in the TSA 100nM condition (red outline) is shown. The 10 commonest indels for each site are shown.

B. As for Fig. 7B, the $\log_2$ fold change in frequency for the indicated indels is shown for the remaining 4 target sites. The 10 commonest indels across both replicates are shown.

Table S5: Precision and Insertion rate associated with dinucleotides at -5 and -4 positions. Related to Figure 5
Dinucleotides represented by more than 10 sites are shown.

-5	-4	Median commonest frequency	Precise %	Middle %	Imprecise %	Insertion %	Deletion %	Number of sites
A	T	0.65	61.5	7.7	30.8	92.3	7.7	13
N	T	0.56	51.2	32.6	16.3	90.7	9.3	43
C	A	0.53	56.3	31	12.7	80.3	19.7	71
C	T	0.45	47.1	47.1	5.9	100		17
N	A	0.42	35	39.9	25.2	77.3	22.7	163
A	A	0.41	26	54	20	84	16	50
C	C	0.39	25	51.4	23.6	30.6	69.4	72
N	C	0.31	13.1	48.3	38.6	44.3	55.7	176
G	C	0.27	6.2	46.9	46.9	62.5	37.5	32
A	C	0.27	4.8	50	45.2	66.7	33.3	42
G	A	0.26	9.4	40.6	50	62.5	37.5	32
C	G	0.25	14.3	33.9	51.8	23.2	76.8	56
T	G	0.25	9.8	35.3	54.9	13.7	86.3	51
T	C	0.24	3.3	40	56.7	26.7	73.3	30
G	G	0.21	9.8	25.5	64.7	19.6	80.4	51
N	G	0.21	7.5	32.6	59.9	21	79	267
A	G	0.21	1.8	33.9	64.2	23.9	76.1	109
