# Supplementary material for: Target-Specific Precision of CRISPR-Mediated Genome Editing
Source: Mol Cell. 2019 Feb 21;73(4):699–713.e6. doi: 10.1016/j.molcel.2018.11.031 (PMC6395888; doi:10.1016/j.molcel.2018.11.031)
Supplement: Document S2. Article plus Supplemental Information [file mmc8.pdf]

Molecular Cell

Article

Target-Specific Precision of CRISPR-Mediated Genome Editing

Graphical Abstract

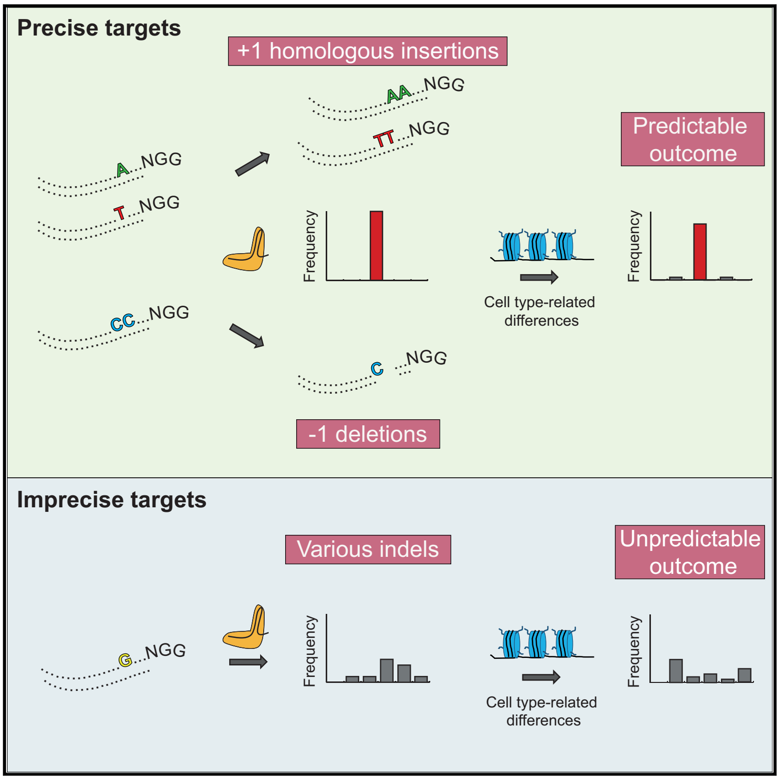

Authors

Anob M. Chakrabarti, Tristan Henser-Brownhill, Josep Monserrat, Anna R. Poetsch, Nicholas M. Luscombe, Paola Scaffidi

Correspondence

arpoetsch@gmail.com (A.R.P.), paola.scaffidi@crick.ac.uk (P.S.)

In Brief

Chakrabarti, Henser-Brownhill, Monserrat et al. show that the genome-editing outcome can be predicted based on simple rules that mainly depend on the target site sequence. Since editing precision varies considerably across sites, careful selection of a predictable target is critical to induce a desired modification in a cell-type-independent manner.

Highlights

- The outcome of CRISPR-mediated editing can be predicted
- Not all target sites are edited in a predictable manner
- The precision of DNA editing is mainly determined by the fourth nucleotide upstream of the PAM site
- Chromatin states affect editing of imprecise, but not precise, target sites

Chakrabarti et al., 2019, Molecular Cell *73*, 699–713
February 21, 2019 © 2018 The Author(s). Published by Elsevier Inc.
https://doi.org/10.1016/j.molcel.2018.11.031

CellPress

Target-Specific Precision of CRISPR-Mediated Genome Editing

Anob M. Chakrabarti,[1,2,6] Tristan Henser-Brownhill,[3,6] Josep Monserrat,[3,6] Anna R. Poetsch,[1,2,4,*] Nicholas M. Luscombe,[1,2,4] and Paola Scaffidi[3,5,7,*]
[1]Bioinformatics and Computational Biology Laboratory, The Francis Crick Institute, 1 Midland Road, London NW1 1AT, UK
[2]UCL Genetics Institute, Department of Genetics, Evolution and Environment, University College London, London WC1E 6BT, UK
[3]Cancer Epigenetics Laboratory, The Francis Crick Institute, 1 Midland Road, London NW1 1AT, UK
[4]Okinawa Institute of Science and Technology Graduate University, Onna-son, Okinawa, Japan
[5]UCL Cancer Institute, University College London, London WC1E 6DD, UK
[6]These authors contributed equally
[7]Lead Contact
*Correspondence: arpoetsch@gmail.com (A.R.P.), paola.scaffidi@crick.ac.uk (P.S.)
https://doi.org/10.1016/j.molcel.2018.11.031

SUMMARY

The CRISPR-Cas9 system has successfully been adapted to edit the genome of various organisms. However, our ability to predict the editing outcome at specific sites is limited. Here, we examined indel profiles at over 1,000 genomic sites in human cells and uncovered general principles guiding CRISPR-mediated DNA editing. We find that precision of DNA editing (i.e., recurrence of a specific indel) varies considerably among sites, with some targets showing one highly preferred indel and others displaying numerous infrequent indels. Editing precision correlates with editing efficiency and a preference for single-nucleotide homologous insertions. Precise targets and editing outcome can be predicted based on simple rules that mainly depend on the fourth nucleotide upstream of the protospacer adjacent motif (PAM). Indel profiles are robust, but they can be influenced by chromatin features. Our findings have important implications for clinical applications of CRISPR technology and reveal general patterns of broken end joining that can provide insights into DNA repair mechanisms.

INTRODUCTION

The CRISPR-Cas9 system has quickly become the preferred tool for genome engineering, enabling site-specific alterations in a variety of organisms and cellular contexts (Hsu et al., 2014). The system relies on the combined use of the bacterial Cas9 endonuclease and a single-guide RNA (sgRNA) to substitute, insert, or delete DNA sequences in almost any desired location in the genome (Hsu et al., 2014). Regardless of the experimental setting and application, genome editing by the CRISPR-Cas9 system entails three steps: (1) scanning of the genome by the RNA-guided Cas9 nuclease (RGN) to find the DNA sequence complementary to the sgRNA, (2) creation of a DNA double-strand break (DSB) by Cas9, and (3) repair of the lesion by the endogenous DNA repair machinery (Hsu et al., 2014). Both the accuracy and efficiency of the processes involved in each of these steps strongly affect the outcome of CRISPR-mediated editing and consequently the utility of the technology. Since the adaptation of the CRISPR system as an engineering tool, several studies have provided insights into the mechanisms affecting CRISPR-mediated DNA editing and have improved the method (Brinkman et al., 2018; Henser-Brownhill et al., 2017; Horlbeck et al., 2016; Hsu et al., 2014; Isaac et al., 2016; van Overbeek et al., 2016; Tsai et al., 2015; Uusi-Mäkelä et al., 2018). However, fundamental questions about how the mammalian genome and proteins interact with Cas9 and the sgRNAs and how cells respond to CRISPR-induced DNA damage remain unanswered. Increasing our knowledge of the mechanisms regulating these interactions is crucial to maximize the potential and safety of CRISPR-based approaches.

A key prerequisite for a good editing tool is the ability to discriminate between on-target and homologous off-target sites. Characterization of selected sgRNAs using both *in vitro* and cellular assays has provided important information about parameters influencing RGN specificity identifying the seed region of guide RNAs (the 10- to 12-nt sequence adjacent to the protospacer adjacent motif [PAM] sequence) as critical for recognition of target sequences (Hsu et al., 2014). This characterization has guided sgRNA-designing algorithms and improved CRISPR fidelity. However, systematic investigation of off-target cleavage sites has shown that predicting the specificity of any given RGN is not straightforward and has revealed that our understanding of how RGNs scan the mammalian genome is incomplete (Tsai et al., 2015). Importantly, by showing that truncated guide RNAs (17–18 nt) exhibit substantially reduced off-target DSBs, this large-scale analysis has proposed modifications that can considerably improve the technology and benefit various applications (Tsai et al., 2015). This example illustrates how systematic characterization of CRISPR-induced alterations in experimental systems may provide information about how RGNs interact with complex genomes and help optimize editing outcome.

This is an open access article under the CC BY license (http://creativecommons.org/licenses/by/4.0/).

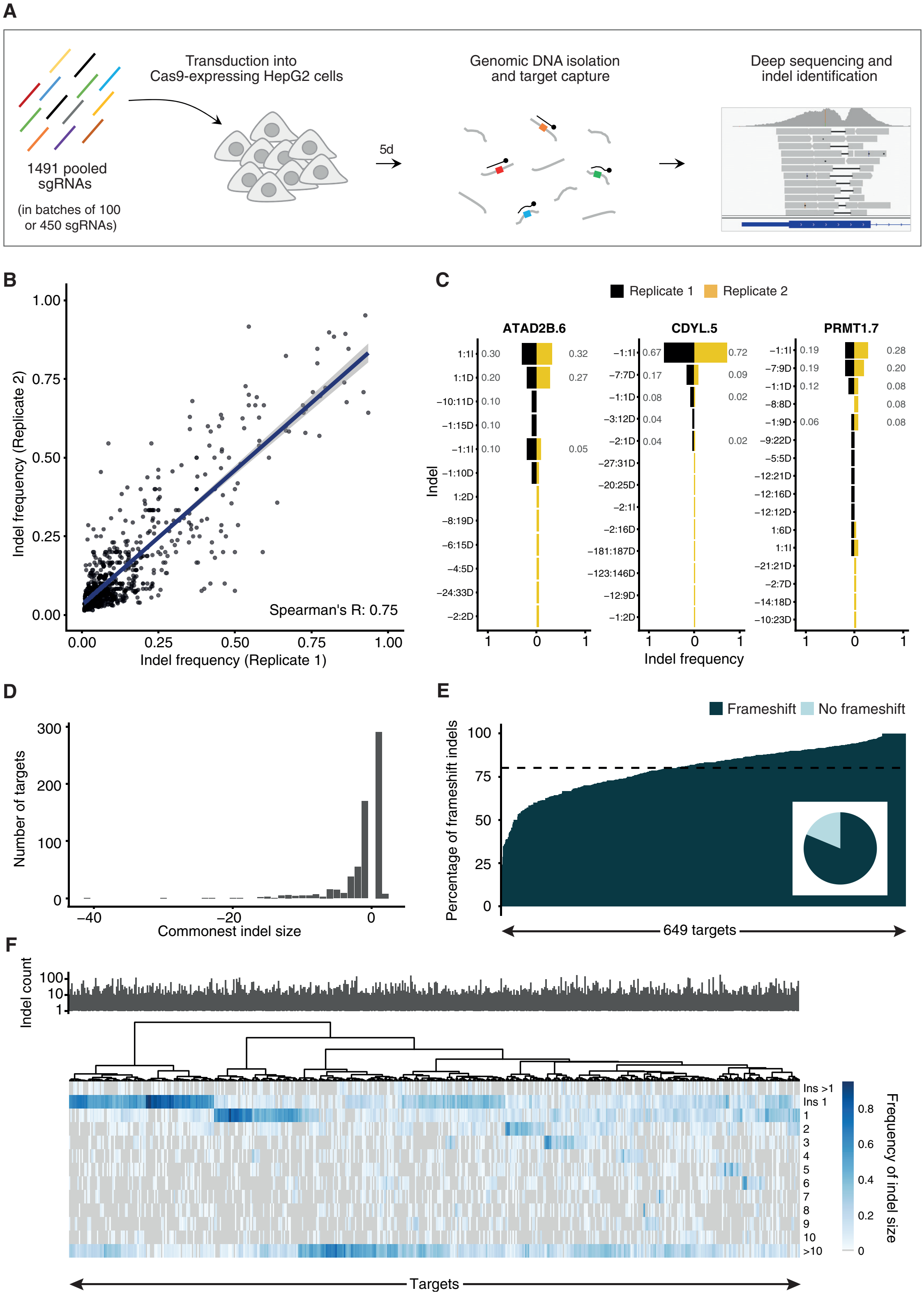

Figure 1. General Specificity and Reproducibility of CRISPR-Mediated Indel Profiles

(A) Overview of the experimental setup.

(B) Frequency at which each detected indel occurs at each target site in two biological replicates.

(legend continued on next page)

In addition to specificity, activity is another feature that can vary widely across RGNs. While direct measurement of cleavage activity at a given target is not simple, sgRNA efficacy has been inferred either by quantifying the frequency of insertion and/or deletion (indel) formation or by evaluating the ability of an sgRNA to induce an expected phenotype. Analysis of large-scale studies has revealed sequence patterns correlating with sgRNA activity and has guided refinement of algorithms for sgRNA design (Doench et al., 2016; Wang et al., 2014). Although *in silico* predictions of sgRNA efficacy have improved considerably, concordance between predicted and empirically measured indel activity remains moderate (Henser-Brownhill et al., 2017). Thus, while we have achieved a qualitative understanding of RGN activity determinants, additional parameters not included in the current algorithms likely contribute to the overall outcome. The epigenetic status of target sequences may be one such factor. Although correlative evidence and *in vitro* studies have implicated chromatin in the modulation of RGN activity (Horlbeck et al., 2016; Uusi-Mäkelä et al., 2018), formal demonstration that the chromatin status of an endogenous locus affects its editing potential is still lacking.

DSBs induced by RGNs at target sites are recognized by the cell's DNA damage response pathways and repaired. Failure of accurate repair creates a chance for sequence alteration. When an exogenous repair template is provided, the homologous recombination (HR) repair pathway allows introduction of precise modifications in the DNA sequence, including single point mutations or insertion of exogenous sequences (Hsu et al., 2014). In the absence of a template, RGN-induced DSBs are often repaired through relatively error-prone mechanisms that result in insertions or deletions of variable length. Indels disrupting gene open reading frames lead to production of truncated, often nonfunctional proteins, making RGN-induced editing an effective means to induce gene knockout (KO) (Hsu et al., 2014). Despite the wide use of the CRISPR system to generate KO alleles, our understanding of the mechanisms driving indel formation is still limited, making the functional outcome of genome editing unpredictable and often preventing a rational use of the technology. Based on the type of indels observed upon RGN-mediated editing, two major repair pathways have been implicated in the formation of RGN-induced indels: canonical non-homologous end joining (cNHEJ), which is known to induce small indels, and microhomology-mediated end joining (MMEJ), which typically generates larger deletions at regions of microhomology (MH) (Deriano and Roth, 2013). Of note, genetic studies examining the general role of these pathways in the formation of CRISPR-mediated indels are currently lacking and the predominant method of repair of RGN-induced DSBs remains unclear. Based on the assumption that NHEJ is the main pathway involved in CRISPR-mediated indel formation, repair outcome was thought to be random. However, recent characterization of indel patterns at multiple genomic locations revealed that individual targets show reproducible repair outcome, with distinct preferences for class (insertion or deletion) and size of indels (van Overbeek et al., 2016). This finding suggests a deterministic nature of RGN-induced break repair and raises questions about the factors involved in defining these nonrandom patterns. Here, we performed a large-scale genomic characterization of indel patterns examining over 1,000 sites in the genome of human cells, with the aim of understanding how genetic and epigenetic factors influence CRISPR-mediated DNA editing. We find that Cas9-induced DSBs are repaired in a predictable or unpredictable way, depending on the target site. Precise targets, which show a dominant indel, can be identified *in silico* and their likely repair outcome inferred by their DNA sequence. Our findings suggest that selection of a predictable target is an effective strategy to induce desired CRISPR-mediated alterations.

RESULTS

Large-Scale Analysis of Indel Patterns

To characterize general patterns of RGN-induced indels, we selected 1,491 target sites across the genome and retrieved the corresponding sgRNAs from a previously generated arrayed lentiviral library (Table S1) (Henser-Brownhill et al., 2017). The library targets 450 nuclear genes with multiple sgRNAs and has shown overall high activity (Henser-Brownhill et al., 2017). At least three sites for each gene were selected, spacing the target regions along genes (Figure S1A) and using sgRNAs with high predicted activity (Chari et al., 2017; Doench et al., 2016) (Figure S1B). Retrieved sgRNAs were combined and sequenced to confirm homogeneous representation in the resulting pools (STAR Methods) (Figures 1A and S1C). Pooled sgRNAs were then transduced into HepG2 cells expressing Cas9 and allowed to edit their target sites for 5 days, a time frame sufficient to reach a plateau in terms of generated indels (Brinkman et al., 2018; van Overbeek et al., 2016) (Figure S1D) but short enough to avoid KO-induced phenotypic changes that may confound the results (Figure S1E). Upon isolation of genomic DNA, target regions were captured by pull-down using custom probes and sequenced at ~6,000- to 8,000-fold coverage (Figures 1A, S2A, and S2B). As expected, infection with pooled sgRNAs resulted in a high proportion of cells with unedited sequence at each target site, since only a small fraction of cells within the population expressed each sgRNA and could edit

(C) Indel profiles for two biological replicates at the indicated target sites. Indel nomenclature: [start coordinate relative to cleavage site]:[size][insertion or deletion]. Counts are normalized to the total library size for each experiment. Numbers in gray indicate indel frequency.

(D) Size distribution of the commonest indel size at each target.

(E) Percentage of indels resulting in a frameshift mutation at each target. Inset pie chart shows the proportion of targets for which the commonest observed indel is a frameshift mutation.

(F) Heatmap visualizing the frequency at which indels of a given size occur at each target. Sites are clustered using Ward D2 hierarchical clustering. The bar plot above indicates the number of indels observed at the corresponding sites. Only data from targets from the 450 pools (524 targets) are used to enable fair comparisons.

See also Figures S1 and S2 and Table S1.

the corresponding site (Figure S2B). Therefore, we developed a custom computational pipeline to filter reads from unedited cells for a given sgRNA, which enabled robust detection of indels (STAR Methods) (Figure S2B). In total, 1,248 sites showed detectable indels, ranging from 1 to 188 per target, with a median count of 32 (Figure S2C). This is a likely underestimation of induced indels, due to the limited sensitivity of our experimental approach, but it provides sufficient repair events to identify general indel patterns. Analysis of target sites in unedited control cells showed minimal indel counts, confirming robust and specific detection of on-target indels (Figures S2C and S2D). Furthermore, high-coverage analysis of cells transduced with individual sgRNAs showed indel profiles very similar to those detected when using pooled sgRNAs (Figure S2E). Targets with at least 10 reads containing indels (649 sites) were selected for downstream analysis.

In agreement with previous studies that examined a limited number of sites (Brinkman et al., 2014; van Overbeek et al., 2016), we observed that RGN-induced editing was highly reproducible across biological replicates (Spearman's coefficient 0.75, $p < 2.2 \times 10^{-16}$), indicating that repair outcome is nonrandom (Figures 1B and 1C). Validated sites confirmed these results, showing almost identical indel patterns in two independent experiments (Figure S2F). Furthermore, our ability to probe a large number of sites simultaneously allowed us to reveal general patterns of CRISPR-mediated DNA editing and make a number of observations. First, single-nucleotide indels were the most frequent type of indel for the majority of targets, with 44% and 26% of targets showing 1-nt insertions or deletions, respectively, as their commonest indel (Figure 1D). Nevertheless, sites showing a preference for longer deletions (up to 41 nt) were also observed (Figure 1D). Second, in line with the observed bias for single-nucleotide alterations, CRISPR-induced indels often resulted in frameshift alterations (Figure 1E). On average, 80.1% of indels induced at a given site disrupted the gene coding frame, a percentage significantly higher than the theoretical 66% assuming a random outcome ($p < 2.2 \times 10^{-16}$, χ^2 test) (Figure 1E). Moreover, 81% of all detected indels resulted in a frameshift (Figure 1E). Thus, the probability of achieving protein loss of function through CRISPR-induced indels is typically relatively high. However, three sites showed strong preference for in-frame indels (in-frame indels $\geq$ 70%), suggesting that in certain cases, it may be difficult to successfully induce gene KO. Third, unsupervised hierarchical clustering identified four groups of targets showing similar indel patterns (Figure 1F). Based on the relative frequency of the observed indels, targets could be broadly divided into sites that preferentially show small insertions, small deletions, long deletions, or have no clear preference (Figure 1F). Fourth, sgRNA activity, as measured by quantifying indel counts at each site, was highly variable, ranging from 0 to 188 (Figures S2C and 1F). Indel count did not correlate with abundance of sgRNAs in the pools, suggesting that sgRNA activity is intrinsically variable (Figure S2G). This observation is in agreement with previous findings obtained by inferring sgRNA activity from their ability to induce an expected phenotype (Doench et al., 2016; Wang et al., 2014). Of note, several inactive sgRNAs had high predicted activity scores, indicating that predicting algorithms can be further improved and that, in addition to DNA sequence, other factors may affect sgRNA activity at a given site (Figure S1B). Activity did not correlate with preference for a certain type of indel pattern (Figure 1F).

Precision of CRISPR-Induced DNA Editing Varies Considerably across Sites

The observation that different targets display distinct preferences for certain indel types prompted us to examine the degree of editing precision (i.e., recurrence of a specific indel) across sites. To do so, we first calculated the relative frequency of each distinct indel, defined by its coordinates and base composition, at each site and then ranked all sites based on the frequency of the commonest indel. This analysis revealed a large range of editing precision, with some targets displaying up to 79 distinct, infrequent indels (frequency < 5%) and others showing one dominant indel (up to 94% frequency) and only a few additional ones (Figures 2A, 2B, and S3A). Overall, we found that for approximately one-fifth of the targets, there is at least a 50% chance of inducing a specific indel, but the majority of sites are more unpredictable. On average, the commonest indel frequency for a given site was 34.1%, and the median number of observed distinct indels was 12.

Editing Precision Correlates with Editing Efficiency, Indel Type, and Indel Size

To examine the relationship between editing precision and indel features, we categorized target sites into three groups: imprecise (0 < commonest indel frequency $\leq$ 0.25), middle (0.25 < commonest indel frequency $\leq$ 0.5) and precise sites (0.5 < commonest indel frequency $\leq$ 1), with each group containing comparable numbers of sites (Figure 3A). Notably, editing precision correlated with efficiency of indel formation ($p < 2.2 \times 10^{-16}$, Kruskal-Wallis test) (Figure 3B). Precise targets showed on average twice as many indels as imprecise targets, and the most active sites showed a strong preference for specific indels (commonest indel frequency > 0.57) (Table S2). This pattern was not due to differences in sgRNA abundance or sequencing depth among groups (Figures S3B and S3C). We then asked whether editing precision correlated with preference for insertions or deletions. Imprecise targets showed a high proportion of deletions, with insertions being on average only 20% of the total indels, whereas insertions were more frequent in the middle group of targets (Figure 3C). Precise targets segregated into two distinct subsets; 68.4% showed a strong preference for insertions, whereas the rest mainly repaired RGN-induced breaks by inducing deletions (Figure 3C). The two subsets were clearly separated, likely reflecting their tendency to induce mainly one dominant indel. Editing precision also correlated with absolute indel size (Figure 3D). While imprecise and middle targets showed a range of indel sizes, with deletions as long as 2,315 bp, precise targets displayed a strong bias toward single-nucleotide indels (Figures 3D, 3E, and S3A). Combining insertion and deletions, 71.5% of edited sequences in the precise group had a single-nucleotide alteration. We conclude that RGN-related editing precision varies considerably across sites and correlates with editing efficiency and the type of resulting indels.

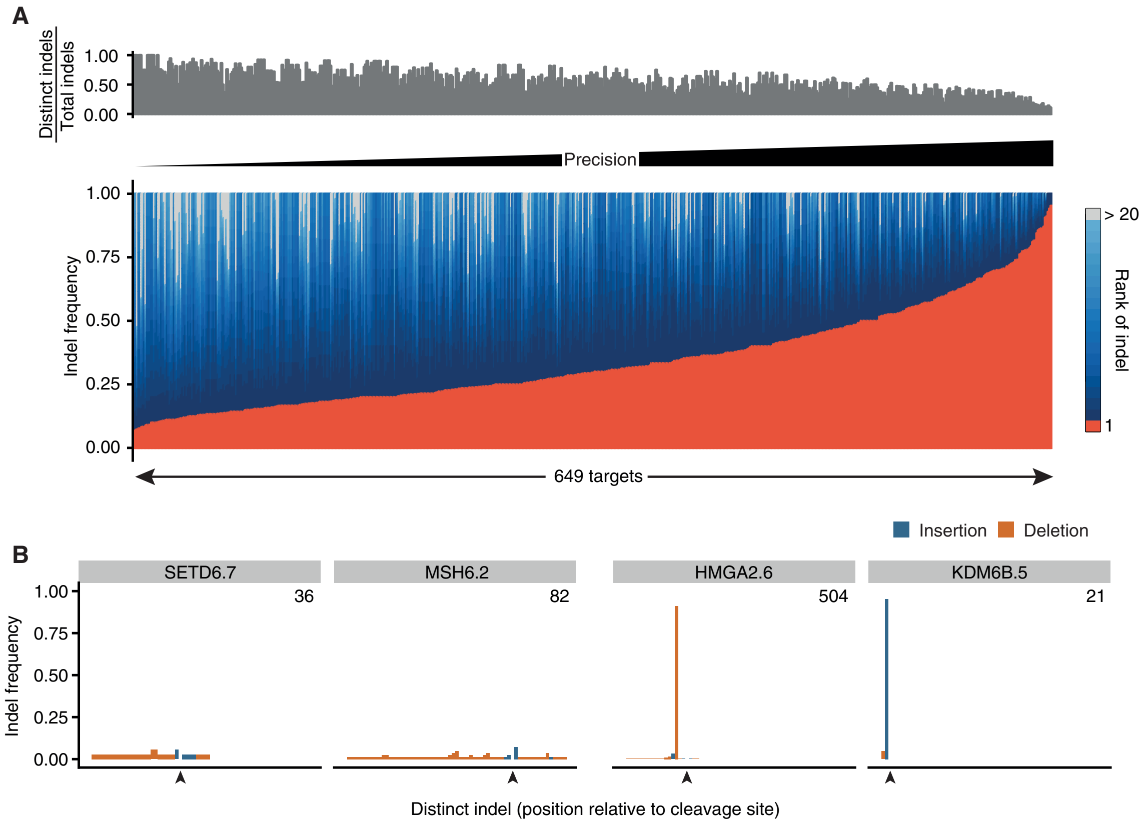

Figure 2. Site-Specific Precision of DNA Editing

(A) Heatmap visualizing the frequency of each indel at each target. Red, commonest indel; blue, indels ranking 2–19; gray, indels ranking higher than 20. Bar plot shows the normalized number of distinct indels at each site.

(B) Indel profiles of two imprecise (left) and two precise (right) targets. Indels are ordered by start coordinate relative to the cleavage site (arrowhead), with insertions having priority over deletions. The inset number indicates the total number of indels detected at that site.

See also Figure S3.

Precise Targets Exhibit Primarily Homology-Associated Insertions and Deletions

Although indel profiles have been shown to be dependent on both MH-dependent and MH-independent mechanisms (Bae et al., 2014; Brinkman et al., 2018; van Overbeek et al., 2016), a quantitative assessment of their relative contribution across many target sites is lacking. In the absence of genetic or pharmacological interference with specific repair pathways (e.g., NHEJ, homology directed repair [HDR], or MMEJ), characterization of indel profiles is insufficient to determine which specific mechanism led to an observed outcome. We therefore performed a pathway-agnostic analysis of indels that searched for any homology at the indel boundaries. This analysis revealed that MH of variable size, ranging from 1 to 18 nt, characterized the majority of deletions (Figures 4A–4C; Table S3). 73.3% of all deletions showed evidence of MH-mediated repair (MH deletions), and on average, 74.3% of deletions at a given site were characterized by MH (Figure 4A). Deletions associated with shorter MHs (1–4 nt) were also enriched above the expected frequency, indicating that the effect of sequence homology on repair outcome is not limited to longer MH stretches (5–25 nt) used by the MHEJ pathway (Figure 4B). MH deletions were enriched in the groups of precise and middle targets ($p = 1.36 \times 10^{-5}$, Kruskal-Wallis test) (Figure 4D). Furthermore, regardless of editing precision, 80% of targets had a MH deletion as their commonest.

Although sequence homology has not been implicated in the formation of insertions, surprisingly, we found that many target sites showed recurrent insertions containing a common inserted base, suggesting that the choice of inserted nucleotide is nonrandom (Figures 4E and S3A; Table S4). Moreover, the recurrently inserted base was often homologous to the nucleotide at position −4 from the PAM sequence, which is typically the nucleotide upstream of the cleavage site (Jinek et al., 2012) (82% of the commonest insertions at each target) (Figure 4F); we termed this feature “insertion homology.” As observed for deletions, the prevalence of insertion homology correlated with editing precision ($p < 2.6 \times 10^{-16}$, Kruskal-Wallis test) (Figures 4G and 4H). Precise targets displayed 96% of homologous insertions, whereas this percentage was only 57% in the imprecise group ($p < 2.6 \times 10^{-16}$, χ^2 test) (Figure 4H), suggesting that template-mediated insertions are a strong determinant of the observed site-specific indel profiles. Even at imprecise targets, homologous insertions were often the commonest ones (Figure 4H). Notably, precise targets showed a strong bias for

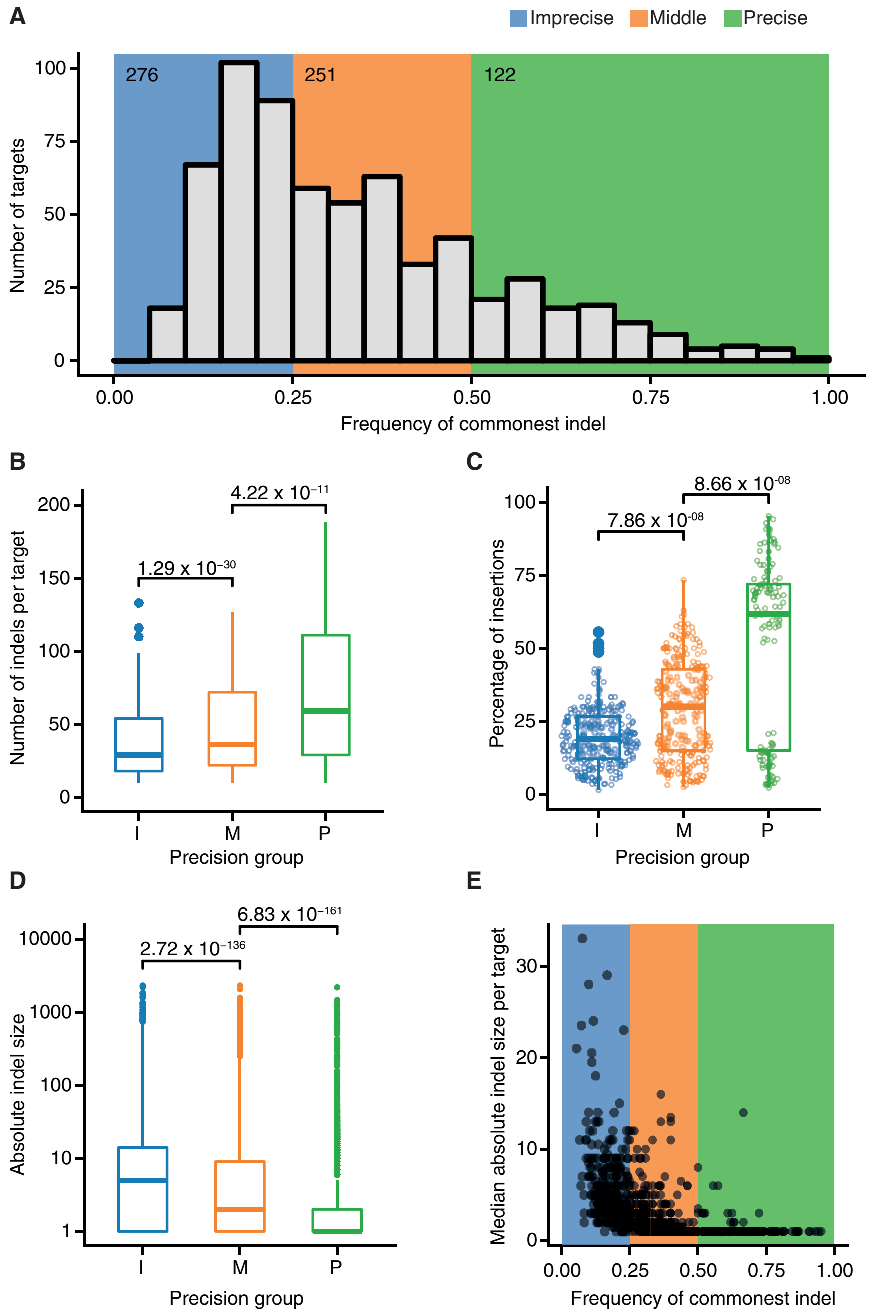

Figure 3. Relationship between Editing Precision and Indel Features

(A) Distribution of commonest indel frequencies at target sites. The background indicates three groups of sites as defined based on their editing precision. Inset numbers indicate the number of target sites in that group.

(B–D) Relationship between precision and indel count (B), type of indel (C), and indel size (D). Only data from the 450 pools are used in (B) to enable fair comparisons.

(C) Percentage of indels that are insertions at each target. I, imprecise; M, middle; P, precise. Statistical analysis was done using the Kruskal-Wallis test followed by Dunn's test for multiple comparisons with Benjamini-Hochberg correction for multiple testing.

(E) Relationship between the median absolute indel size and the commonest indel frequency (i.e., the measure of editing precision at each target). The background is colored as in (A).

See also Figures S3 and Table S2.

inserted "A"s and "T"s, suggesting that sequence features underlie the correlation between editing precision and homologous insertions (Figure 4I). Altogether, these observations suggest that homology-mediated end joining strongly influences DNA repair outcome, for both insertions and deletions, and correlates with site-specific precision of CRISPR-mediated editing.

The DNA Sequence Determines Editing Precision

To examine whether editing precision depends on the base composition of target sites and, if so, to identify critical positions in the protospacer, we employed a machine learning approach. We trained a neural network that predicts editing precision (i.e., commonest indel frequency) using 80% of the targets selected randomly to train the network, with the remaining 20% kept unseen for testing. We found a significant correlation between the estimated and observed indel frequencies for the 130 test target sites (correlation coefficient R = 0.49, p = 4.73 × 10^{-9}, Wald test) (Figures 5A and S4A). Analysis of an independent dataset characterizing indel profiles at 96 distinct sites (van Overbeek et al., 2016) confirmed these findings (R = 0.53, p = 7.26 × 10^{-8}) (Figures 5B and S3E). Importantly, targets analyzed by van Overbeek et al. were selected differently from ours and showed distinct overall nucleotide composition, indicating that the neural network has learned generalizable features (Figures S3D and S4B). Although the predictive power of the model was only moderate (coefficient of determination R^2 = 0.24), it allowed us to identify important positions in the protospacer. If certain positions have a significant influence on editing precision, then randomizing those nucleotides is expected to dramatically reduce the correlation between estimated and observed indel frequencies. To investigate this, we performed a permutation "nucleotide" importance analysis, systematically randomizing each position in test sequences and examining the resulting effect on the neural network output. This analysis revealed that the nucleotide at position −4 from the PAM sequence had the strongest influence on editing precision as a single nucleotide, reducing the model's accuracy by 78% ± 9% upon randomization (R^2 = 0.05 ± 0.02) (Figure 5C). Nucleotide

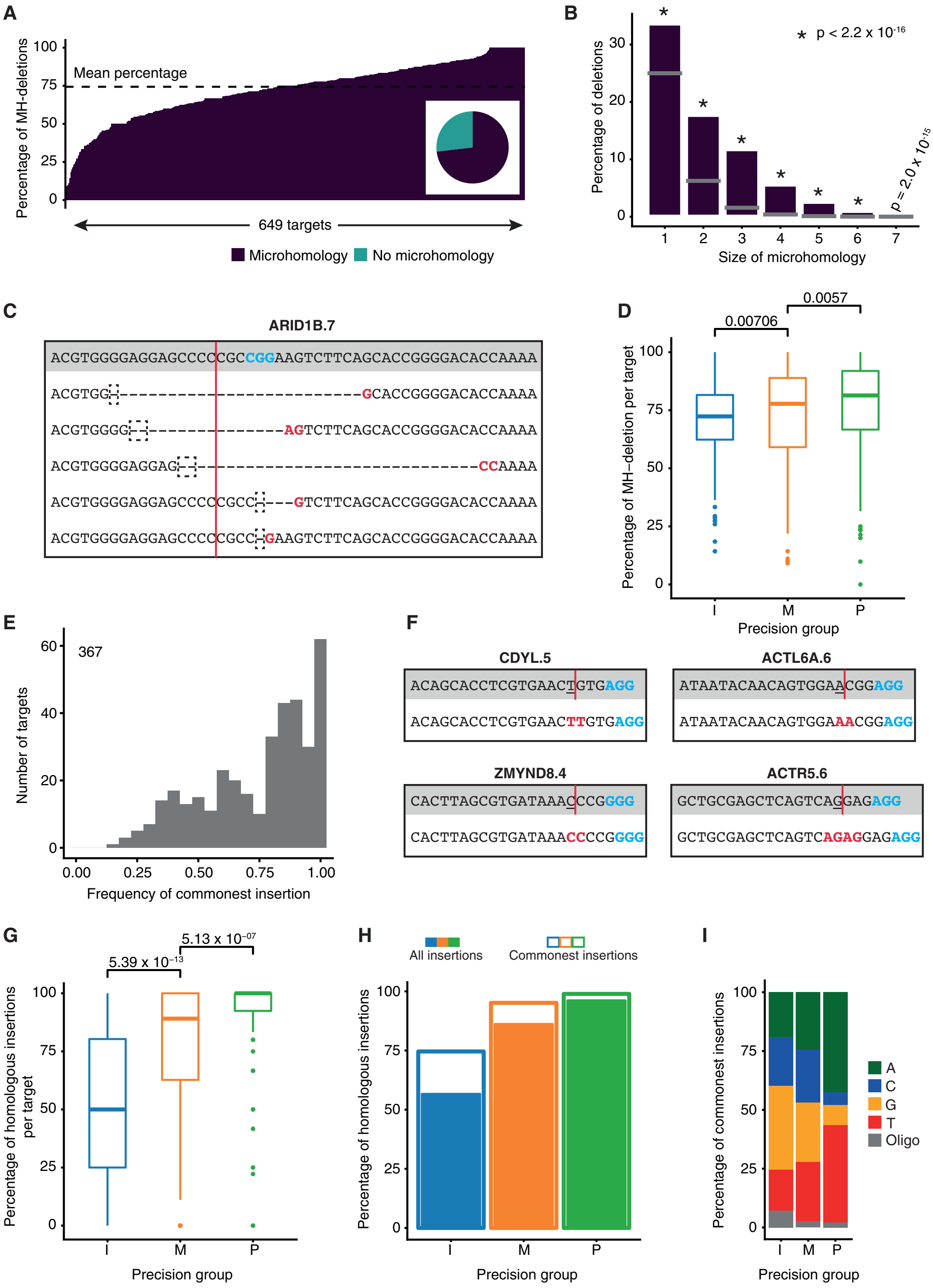

Figure 4. Precise Targets Are Enriched for Homology-Associated Indels

(A) Percentage of microhomology (MH)-associated deletions at each target site. Inset pie chart shows the proportion of all detected MH deletions.

(B) Percentage of deletions that have MH of a given size. The gray bar indicates the expected percentage for each *k*-mer size. Statistical analysis was done using the χ^2 test.

(legend continued on next page)

positions −2, −3, and −5 also showed an effect, although weaker, reducing R^2 by 29% ± 9%, 15% ± 5%, and 50% ± 13%, respectively. Simultaneous randomization of all four nucleotides reduced R^2 by over 98% ± 2% and abolished the predictive significance of the trained model (average R^2 = 0.01 ± 0.01; $p > 0.1$ for all permutations, Wald tests), indicating that these positions within the protospacer, especially the one upstream of the cleavage site, are critical for defining editing precision of a target site (Figure 5D). We refer to these combined nucleotides as the "precision core" of a target site. Similar results were obtained using a least absolute shrinkage and selection operator (LASSO) linear regression model (Figures S4C and S4D).

Targets in different precision groups revealed differences in protospacer nucleotide composition (Figures 5E and S4E). Notably, precise targets showed distinct base preferences depending on whether the commonest indel was an insertion or a deletion (Figure 5E). As expected, nucleotide −4 showed the biggest differences, followed by nucleotide −5, which was frequently a "C," specifically in precise targets (Figure 5E). We then examined to what extent nucleotide −4 on its own could predict editing outcome. Different bases at position −4 showed distinct association with indel types (insertions versus deletions) and precision groups (Figure 5F). The vast majority of target sites that contained an "A" or a "T" upstream of the cleavage site repaired RGN-induced DSBs via insertions (77% and 91% of targets, respectively) (Figure 5G). These were mostly precise or middle targets (median commonest indel frequency: 0.42 and 0.56 for targets with "A" and "T," respectively) (Figures 5G and S4F). When taking into account positions −5 and −4 together, the correlation with precision further increased (median commonest indel frequency: 0.53 and 0.65 for targets with "CA" and "AT," respectively) (Figure 5E; Table S5). In contrast, 79% of targets containing a "G" at position −4 showed deletions and were mostly imprecise targets (median commonest indel frequency: 0.21) (Figures 5G and S4F). Moreover, 76.4% of targets containing "CC" at positions −5 and −4 induced relatively precise deletions (median commonest indel frequency: 0.39) (Figure 5E; Table S5). Notably, similar distributions were observed at the sites edited by van Overbeek et al. (2016) (Figures S4F and S4G). Given the large number of sites examined, the observed percentages assume a predictive value with respect to the editing outcome that may occur at similar protospacers (Figure 5H). We conclude that precise targets can be identified by examining the base composition of the precision core and that position −4 is sufficient to predict with a high degree of confidence whether a site will acquire insertions or deletions.

Chromatin States Affect RGN Activity

Our findings, in agreement with previous small-scale studies (Brinkman et al., 2014; van Overbeek et al., 2016), suggest that DNA sequence features strongly affect RGN-induced indel profiles in a site-specific manner, influencing editing precision and efficiency. However, even within precision groups, the number of induced indels and their patterns varied across sites (Figure 3B). Furthermore, the neural network model, based solely on the protospacer sequence, was unable to fully recapitulate observed frequencies, suggesting other factors at play. We therefore examined whether chromatin structure may contribute to the observed editing outcome. To do so, we selected six target sites characterized by variable editing precision and efficiency of indel formation (Figure 6A) and individually transduced the corresponding sgRNAs in Cas9-expressing cells in the presence of chromatin-modulating compounds. We used the histone deacetylase (HDAC) inhibitor trichostatin A (TSA) to induce histone hyperacetylation at the target sites (Figures S5A and S5B) using concentrations of the inhibitor that do not impair cell proliferation or induce DNA damage (Figures S5C and S5D). TSA treatment significantly increased the efficiency of indel formation, inducing dose-response changes ($p < 0.001$, paired Wilcoxon test) and reaching almost a 2-fold increase for the ACTL6A.5 site (Figure 6B). The effect was highly reproducible across biological replicates (Figures 6B and 6D; Table S6), varied depending on the target, and inversely correlated with the endogenous levels of histone acetylation (Figures 6B, 6C, and S5B). Sites characterized by low levels of acetylated H3 showed a greater response to the treatment than those that already had high levels of the endogenous mark (MSH6.2 and SMARCD2.1), suggesting a direct effect of chromatin modulation on indel formation (Figures 6B, 6C, and S5B). Editing efficiency was also affected, to a lower extent, by treatment of cells with EZH2i inhibitors, which decreased H3K27me3 levels (Figure S5A). Contrary to TSA, EZH2i inhibited indel formation (Figure 6B). Analysis of individual indels indicated that the effect of TSA and EZH2i was not restricted to a few indels and that both insertions and deletions were affected (Figures 6D and S6A; Table S6). We conclude that the chromatin state of target sites affects the

(C) Deletions detected at the ARID1D.7 site. In the gray panel is the reference sequence, with the PAM sequence emboldened in blue and the expected cleavage site indicated with a red line. Below, each line represents a detected deletion. In the dashed box is the MH in the deletion, and emboldened in red is the corresponding MH in the unedited part of the sequence.

(D) Percentage of MH deletions at individual sites grouped by precision. I, imprecise; M, middle; P, precise. Statistical analysis was done using the Kruskal-Wallis test followed by Dunn's test for multiple comparisons with Benjamini-Hochberg correction for multiple testing.

(E) Frequency of the commonest insertion at a target site. Only targets with 5 or more insertions are considered to obviate a low-count bias. The inset count is the number of target sites included.

(F) Insertions detected at the indicated sites. In the gray panel is the reference sequence, with the PAM sequence emboldened in blue and the expected cleavage site indicated with a red line. The −4 position is underlined. Below, the edited sequence is shown with the insertion homology (either a mono- or dinucleotide) emboldened in red.

(G) Percentage of homologous insertions at individual target sites grouped by precision. Statistical analysis was done using the Kruskal-Wallis test followed by Dunn's test for multiple comparisons with Benjamini-Hochberg correction for multiple testing.

(H) Percentage of all homologous insertions in a group (filled bars) and corresponding percentage of commonest insertions (outlined bars).

(I) Nucleotide inserted as the commonest insertion for each precision group.

See also Tables S3 and S4.

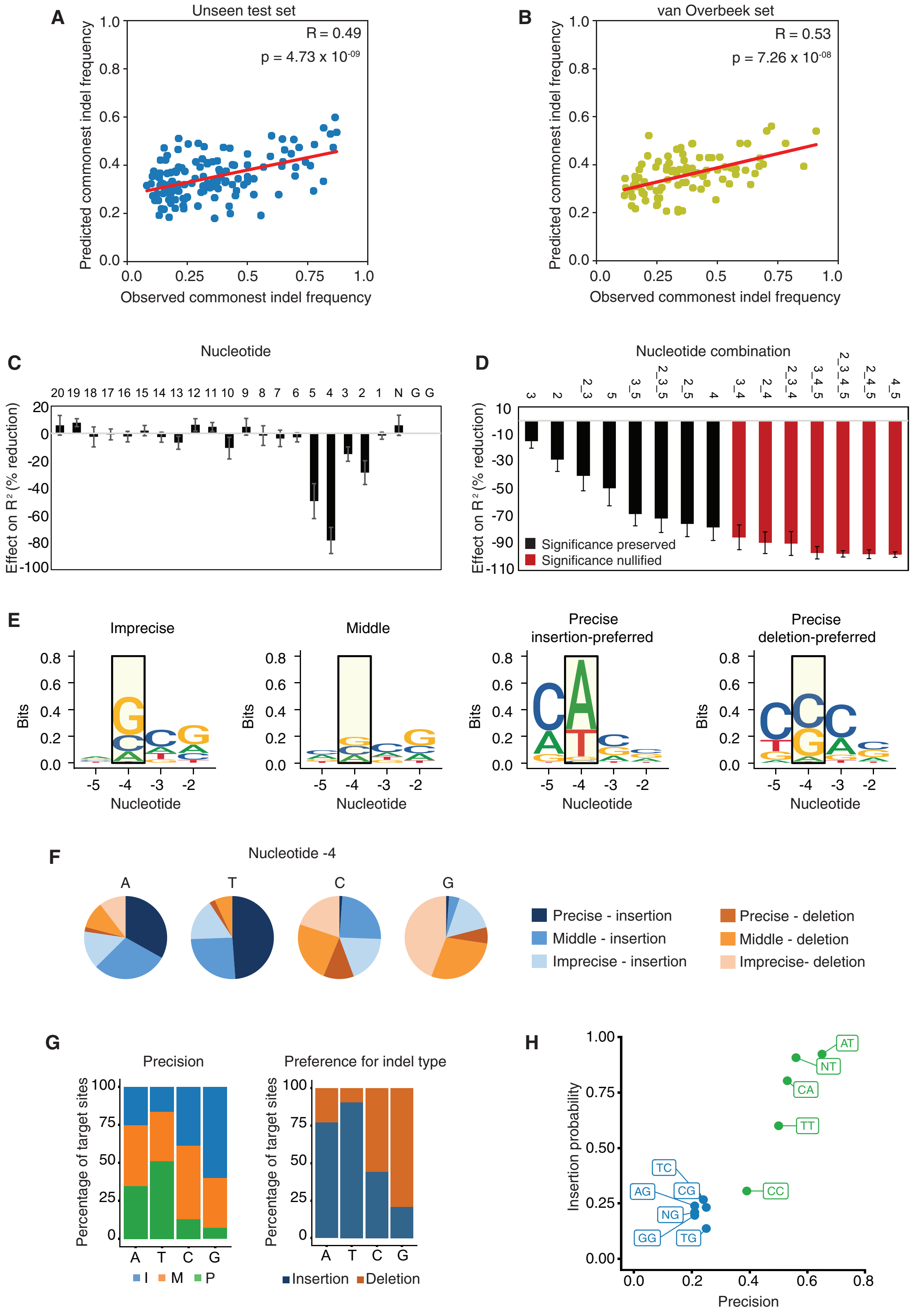

(legend on next page)

activity of RGNs and that transient induction of histone acetylation enhances DNA editing efficiency.

Chromatin States Influence Indel Profiles but Do Not Alter Dominant Indels at Precise Sites

Although changes in editing efficiency by TSA or EZH2i were observed for most indels at each site, some indels were preferentially affected (Figure 6D). Furthermore, shorter and longer indels appeared differentially altered by treatment (Figure S6B). These observations suggest that chromatin modulation may affect indel profiles. We therefore examined the relative changes in the abundance of individual indels, focusing on the effect of TSA, which induced greater and more consistent changes in indel formation (Figures 6B and 6D). Across all sites, we observed dose-dependent changes in the relative frequency of indels, with some being favored at the expense of others (Figures 7 and S7). Although the observed changes were small in extent and the overall indel patterns were maintained, confirming robustness of the editing profiles, the most frequent indels showed reproducible and dose-dependent changes (Figure 7). At some sites (MBD3L1.6, MSH6.2, and SMARCD2.1), the preference for their commonest indel was enhanced, while at others (ACTL6A.5, ASF1B.7, and BRD2.7), it was decreased (Figure 7C). Importantly, changes induced by chromatin modulation had distinct impact on sites, depending on their editing precision; for instance, the identity of the commonest indel changed at the imprecise BRD2.7 site, whereas the dominant indel at the precise ACTL6A.5 site was not altered, despite significant changes in its frequency (Figures 7A and 7C). Thus, editing of precise targets is not substantially affected by differences in chromatin states, whereas dominant indels can vary at imprecise targets depending on chromatin state. This observation has implications for DNA editing in different cell types.

As a complementary approach to experimental modulation of chromatin, we analyzed the van Overbeek dataset, which examined indel profiles at 96 sites in different cell types characterized by distinct chromatin landscapes. HCT116 cells were excluded from this analysis, as their deficiency in mismatch repair may modulate indel profiles independently of chromatin differences. Embryonic kidney HEK293 cells and lymphoblastoid K562 cells displayed very similar but not identical indel profiles, indicating that these are primarily, but not entirely, determined by DNA sequence (Figure S5E). Sites with major differences in histone acetylation levels showed different indel profiles. As observed in our dataset, some imprecise targets showed different dominant indels in the two cell lines, whereas precise sites showed conserved indel profiles (Figure S5F). Altogether, these results show that chromatin structure contributes to the establishment of site-specific indel profiles. While the DNA sequence appears to be the major determinant of CRISPR-mediated editing outcome, the chromatin state of a given site may modulate the relative abundance of individual indels and contributes to defining the site's indel profile. Despite chromatin-mediated differences in indel profiles, precise targets display a conserved and highly reproducible editing outcome.

DISCUSSION

Precision of Editing Outcome

Although the bacterial CRISPR system has been widely adopted as the preferred genome engineering tool, our ability to predict the editing accuracy, efficacy, and outcome at specific sites is still limited. A major obstacle in defining precise genome editing rules is our incomplete understanding of how RGNs interact with eukaryotic cellular components—complex genomes containing repetitive sequences, the packaging of DNA into chromatin, and the presence of various cellular pathways that recognize and repair RGN-induced DSBs. Various studies have provided insights into some of these interactions (Brinkman et al., 2018; Isaac et al., 2016; Jensen et al., 2017; Kosicki et al., 2018; Lemos et al., 2018; van Overbeek et al., 2016). However, due to the limited number of characterized target sites, discerning whether the observed patterns are general or site-specific features is not straightforward. Through systematic analysis of indel formation at over 1,000 different sites in the human genome, this study reveals general trends of CRISPR editing and provides simple rules to predict how a given target may respond to RGN-induced DSBs.

Extending the observation that indel profiles are nonrandom (van Overbeek et al., 2016), we find that precision of DNA editing varies considerably among sites, with some targets showing one highly preferred sequence alteration and others displaying a wide range of infrequent, yet reproducible, indels. We show that editing precision is an intrinsic feature of the target site and depends on four nucleotides located around the cleavage site within the protospacer, with the most influential position being the nucleotide at position −4 from the PAM sequence. Strikingly, the mere presence of a "T" here gives a site a 51%

Figure 5. A Neural Network Identifies Protospacer Nucleotide Positions that Determine Editing Precision

(A and B) Correlation between the observed precision at a given target site and that predicted by the neural network, using our test set (A) and independent dataset (van Overbeek et al., 2016) (B). R, correlation coefficient. Statistical analysis was done using the Wald χ^2 test.

(C and D) Contribution of the indicated protospacer nucleotides (C) or combination of nucleotides (D) to editing precision. The effect of nucleotide randomization is shown as reduction of the model's accuracy (R^2). Values are mean and SD from 10 different permutations. Bars in red indicate randomized positions that increased p values of Wald tests across the majority of permutations to nonsignificant levels ($p > 0.05$).

(E) Sequence logos for the precision core for the different precision groups. Precise targets are split based on their preference (commonest indel) for insertions or deletions. The most important −4 nucleotide position is highlighted in a yellow box.

(F and G) Proportion (F) and percentage (G) of targets that have the indicated nucleotide at the −4 position. Sites are grouped based on their precision and their preference (commonest indel) for insertions or deletions.

(H) Likelihood of editing outcome for sites having the indicated nucleotides at the −5 and −4 positions. Numbers represent the median commonest frequency and the insertion rate for each mono- or dinucleotide as measured in our dataset. See also Table S5.

See also Figure S4 and Table S5.

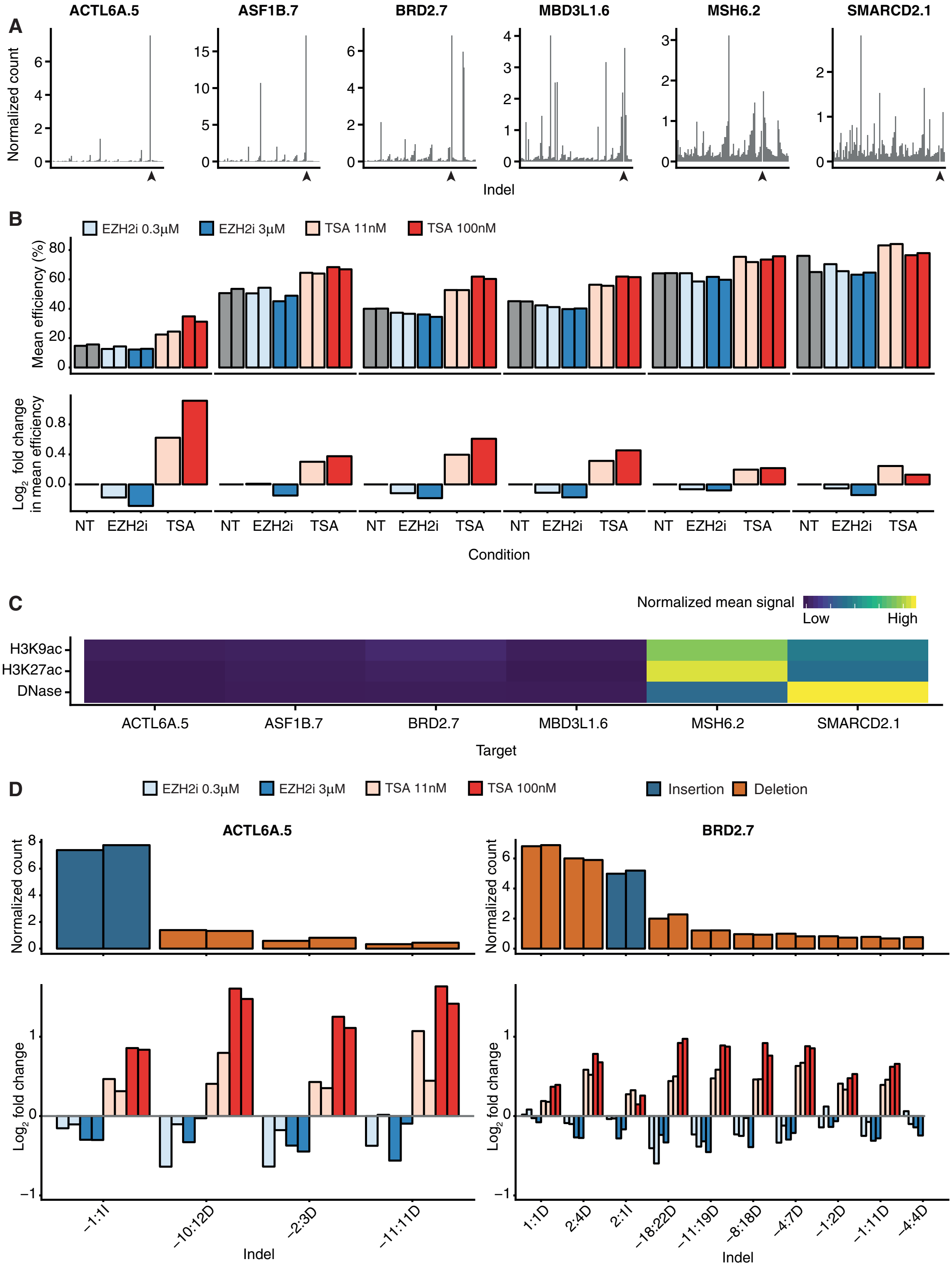

Figure 6. Chromatin Modulation Affects RGN Activity

(A) Indel profile at the indicated target sites in untreated cells. Indels are ordered by start coordinate relative to the cleavage site (arrowhead), with counts normalized by the effective library size at each site. The mean across both replicates is shown.

(B) Editing efficiency (above) and $\log_2$ fold-change in efficiency relatively to untreated cells (NT) (below) for each target site in the indicated conditions. Biological replicates are shown separately in the upper graphs and averaged in the bottom graphs.

(legend continued on next page)

probability of repairing in a predictable manner and 91% chance of introducing an insertion. Our finding that editing precision is site-specific and can be predicted has important implications. Practically, knowing what editing outcome is likely to occur at a given site maximizes the chance of having a desired sequence alteration, for both clinical and research applications. Although pharmacological modulation of repair pathways alters indel profiles, the induced changes are subtle, and for many applications, the use of inhibitors may not be suitable (van Overbeek et al., 2016; Shou et al., 2018). Targeting a precise site would be a more effective way of steering CRISPR-mediated editing toward a desired outcome. Moreover, given the extreme reproducibility of indel patterns, the selection of a precise target combined with experimental validation in model systems could considerably increase safety in clinical applications. This is particularly relevant in light of recent studies reporting the occurrence of large on-target deletions that may have pathological consequences (Kosicki et al., 2018).

Relationship between Editing Precision and Indel Type

Our findings also reveal a strong correlation between editing precision and preference for repairing RGN-induced DSBs via insertions. We show that targets with "A"s or "T"s at nucleotide −4 mainly show insertions, with the commonest insertion being highly recurrent and representing on average approximately half of the indels detected at a given site (Figure 5H). DSB repair via insertions may be kinetically faster compared to other types of indel, partly explaining the higher efficiency of precise targets and the general bias toward single-nucleotide indels. Notably, recent studies have reached similar conclusions using experimental approaches complementary to ours, based on synthetic target sites (Allen et al., 2018; Shen et al., 2018; Taheri-Ghahfarokhi et al., 2018). The identity of the recurrent insertions can also be predicted, as the inserted nucleotide is nearly always homologous to the −4 nucleotide (Figures 4G–4I). Such predictions could, for instance, allow efficient introduction of a stop codon (TAA) when an in-frame TA dinucleotide is present at positions −5 and −4 of the targeted region. In contrast, targets with "G"s at nucleotide −4 are the most imprecise and repair mainly induces a variety of unpredictable deletions (Figures 5G and 5H). Thus, choosing target sites with "A"s or "T"s at nucleotide −4 is an effective way to induce predictable insertions at regions of interest.

Critical Role of Nucleotide –4 in Defining Site-Specific Indel Profiles

The key role of nucleotide −4 in influencing editing precision and preference for indel type is particularly interesting in light of recent findings that revealed flexible scissile profiles by Cas9 and generation of 5′ overhangs upstream of the canonical cleavage site due to asymmetric cleavage of the two DNA strands (Shou et al., 2018). Notably, 5′ overhangs are mostly observed at position −4 on the non-complementary strand. These findings, together with our results, explain the prevalence of single-nucleotide insertions homologous to the −4 nucleotide, as the overhanging nucleotide can be used as a template before ends are rejoined. Thus, paradoxically, imprecision of Cas9 cleavage is the likely cause of precision in the insertion outcome. Similarly, the high frequency of single-nucleotide deletions is likely related to the asymmetric cleavage of DNA by Cas9.

Envisioning how the base composition of position −4 may influence editing precision is not straightforward. One possibility is that the nature of the 5′ overhanging nucleotide may recruit distinct proteins involved in DNA repair. Alternatively, it may affect Cas9 binding to the broken ends, and this may, in turn, affect the repair outcome. The other nucleotides in the precision core may act similarly. Structural analysis of RGNs with distinct −4 nucleotides may help shed light on this issue.

Our observation that the vast majority of detected insertions show homology, combined with the finding that NHEJ-mediated repair of CRISPR-induced DSBs is mostly error-free (Geisinger et al., 2016) and that deletions generated by sgRNA pairs can be repaired with a high level of precision (Shou et al., 2018), suggests a model whereby flexible cleavage by Cas9 influences DNA repair fidelity; when blunt ends are generated at nucleotide −3, cells repair DSBs in an error-free manner, reconstituting the original sequence, whereas indels occur mainly when asymmetric cleavage generates overhanging ends. This model may also reconcile apparently conflicting results about the fidelity of NHEJ in CRISPR-independent and CRISPR-dependent contexts (Brinkman et al., 2018; Dudley et al., 2005; Geisinger et al., 2016; van Heemst et al., 2004; Shou et al., 2018). Interestingly, both outcomes are useful for genome editing, as blunt ends allow precise genomic deletions and insertions of exogenous sequences, while overhanging ends enable induction of indels resulting in gene KO.

Influence of the Chromatin Environment on Site-Specific Editing Outcome

Although DNA sequence is a major determinant of site-specific indel profiles, we show that packaging of DNA into chromatin may affect editing efficiency and the relative frequency of indels at a given locus. We find that histone hyperacetylation and reduction of the heterochromatin-associated mark H3K27me3 induce opposite changes in editing efficiency, enhancing and inhibiting indel formation, respectively. Although the effect of TSA was observed at all tested sites, the effect was particularly pronounced at sites with low endogenous levels of histone acetylation, suggesting that transient TSA treatment may be a

(C) Mean chromatin immunoprecipitation sequencing (ChIP-seq) signal for H3K9ac and H3K27ac and DNase-seq signal in untreated HepG2 cells (Kundaje et al., 2015). Signal in a 500-nt window centered on the cleavage site at each target site is shown as a heatmap.

(D) Chromatin modulation affects both insertions and deletions. Count of individual indels at the indicated sites in untreated cells (above), and $\log_2$ fold-change in efficiency induced by TSA or EZH2i relative to untreated cells (below). Indel count is normalized to the effective library size at each site for each replicate. Only indels with a normalized count of at least 1 in any condition are included. The indel nomenclature is [start coordinate relative to cleavage site]:[size][insertion or deletion].

See also Figures S5 and S6 and Table S6.

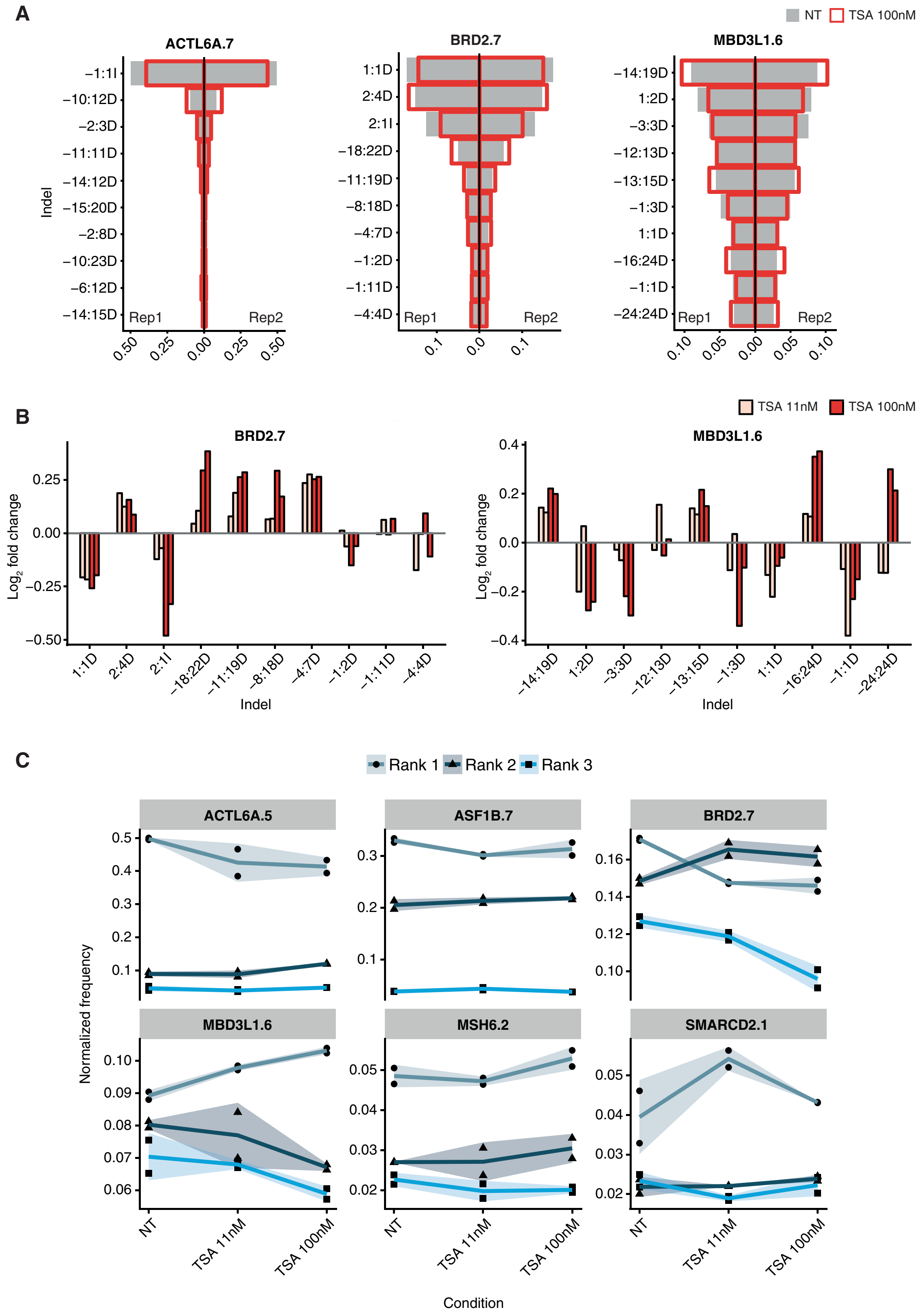

(legend on next page)

strategy to enhance editing efficiency at sites located in repressive chromatin environments. While our results do not unequivocally prove that local chromatin changes are responsible for the observed effects, they are in agreement with the reported correlations between sgRNA activity and open chromatin at the genome-wide levels and evidence from *in vitro* studies indicating that nucleosome positioning impairs binding of Cas9 to DNA and inhibits its activity (Horlbeck et al., 2016; Uusi-Mäkelä et al., 2018). In addition to interfering with Cas9 binding to a target site, chromatin may also affect its cleavage profile, favoring either blunt ends that can be precisely repaired or overhanging ends that promote the formation of indels. We also show that modulation of chromatin differentially affects individual indels at a target site and can change the identity of the commonest indel at imprecise sites (Figure 7). Notably the magnitude of changes observed upon TSA treatment, albeit small, is comparable to those observed when inhibitors of specific DNA repair pathways are used (van Overbeek et al., 2016). These results show that the chromatin configuration of a given site contributes to defining its indel profile. Given the established role of chromatin in DNA repair (Kalousi and Soutoglou, 2016) and the involvement of multiple DNA repair pathways in mediating CRISPR-induced DNA editing (Maruyama et al., 2015; van Overbeek et al., 2016; Shou et al., 2018), altered recruitment of factors involved in different pathways may underlie the observed difference upon chromatin modulation. Importantly, regardless of chromatin states, precise targets show consistent dominant indels, suggesting that editing outcome at these sites is conserved across cell types.

In summary, our findings uncover general principles guiding CRISPR-mediated DNA editing in human cells and provide guidelines for a more effective and safer use of the technology, with important implications for clinical applications. They also reveal a striking influence of the DNA sequence in dictating DSBs repair outcomes and lay the foundation for future mechanistic studies that can increase our understanding of end-joining processes in human cells.

STAR★METHODS

Detailed methods are provided in the online version of this paper and include the following:

- KEY RESOURCES TABLE
- CONTACT FOR REAGENT AND RESOURCE SHARING
- EXPERIMENTAL MODEL AND SUBJECT DETAILS
  - Cell lines
- METHOD DETAILS
  - sgRNAs pool generation
  - Viral transductions
  - Timing of CRISPR-mediated editing
  - Protein detection
  - Chromatin modulation and ChIP-qPCR
  - Cell proliferation
  - Library preparation and deep sequencing
  - Sequencing read alignment
  - Indel identification
  - Characterization of target sites
  - Analysis of van Overbeek data
  - Indel profiles upon chromatin modulation
  - Analysis of chromatin environment
  - Analysis of nucleotide influence
- QUANTIFICATION AND STATISTICAL ANALYSIS
- DATA AND SOFTWARE AVAILABILITY

SUPPLEMENTAL INFORMATION

Supplemental Information includes seven figures and seven tables and can be found with this article online at https://doi.org/10.1016/j.molcel.2018.11.031.

ACKNOWLEDGMENTS

We thank the Crick Advanced Sequencing and Bioinformatics and Biostatistics facilities for preparing and sequencing NGS libraries and for help with data processing. We thank Her Majesty Queen Elizabeth II for starting the sequencing run containing our samples and Andrew J. Steele for comments on the machine learning analysis. This work was supported by the Francis Crick Institute, which receives its core funding from Cancer Research UK (grants FC001110 and FC001152); the UK Medical Research Council (grants FC001110 and FC001152); and the Wellcome Trust (grants FC001110 and FC001152), and by the CRUK Drug Discovery Award (C50796/A19448) to P.S. N.M.L. is a Winton Group Leader in recognition of the Winton Charitable Foundation's support towards the establishment of the Francis Crick Institute. This work was also supported by a Wellcome Trust PhD Training Fellowship for Clinicians Award (110292/Z/15/Z) to A.M.C. and a postdoctoral fellowship by the Peter and Traudl Engelhorn Foundation to A.R.P.

AUTHOR CONTRIBUTIONS

A.M.C. performed most of the computational analysis and wrote the manuscript with P.S. T.H.-B. generated all reagents used in the study and performed the large-scale experiment and ANN analysis. J.M. performed the chromatin modulation experiments. A.R.P. and N.M.L. supervised the computational work and provided input on the manuscript. P.S. conceived the study, analyzed the data, supervised the work, and wrote the manuscript.

DECLARATION OF INTERESTS

The authors declare no competing interests.

Received: July 30, 2018
Revised: October 25, 2018
Accepted: November 20, 2018
Published: December 13, 2018

Figure 7. Chromatin Modulation Induces Small Changes in Indel Profiles
(A) Normalized indel frequency for the indicated targets in untreated cells (gray bars) and in cells treated with 100 nM TSA (red outline). Indel nomenclature: [start coordinate relative to cleavage site]:[size][insertion or deletion]. The 10 commonest indels for each site are shown.
(B) Log_2 fold change in the indel frequency for the indicated targets. The 10 commonest indels across both replicates are shown.
(C) Change in frequency for the three commonest indels (ranks 1, 2, and 3) for all validated target sites. The line indicates the mean of both replicates, and the shaded area represents the mean ± 1 SD. NT, untreated cells.
See also Figures S5 and S7 and Table S6.

REFERENCES

Allen, F., Crepaldi, L., Alsinet, C., Strong, A.J., Kleshchevnikov, V., De Angeli, P., Páleníková, P., Khodak, A., Kiselev, V., Kosicki, M., et al. (2018). Predicting the mutations generated by repair of Cas9-induced double-strand breaks. Nat. Biotechnol. https://doi.org/10.1038/nbt.4317.

Bae, S., Kweon, J., Kim, H.S., and Kim, J.-S. (2014). Microhomology-based choice of Cas9 nuclease target sites. Nat. Methods *11*, 705–706.

Brinkman, E.K., Chen, T., Amendola, M., and van Steensel, B. (2014). Easy quantitative assessment of genome editing by sequence trace decomposition. Nucleic Acids Res. *42*, e168.

Brinkman, E.K., Chen, T., de Haas, M., Holland, H.A., Akhtar, W., and van Steensel, B. (2018). Kinetics and fidelity of the repair of Cas9-induced double-strand DNA breaks. Mol. Cell *70*, 801–813.e6.

Chari, R., Yeo, N.C., Chavez, A., and Church, G.M. (2017). sgRNA Scorer 2.0: a species-independent model to predict CRISPR/Cas9 activity. ACS Synth. Biol. *6*, 902–904.

Deriano, L., and Roth, D.B. (2013). Modernizing the nonhomologous end-joining repertoire: alternative and classical NHEJ share the stage. Annu. Rev. Genet. *47*, 433–455.

Doench, J.G., Fusi, N., Sullender, M., Hegde, M., Vaimberg, E.W., Donovan, K.F., Smith, I., Tothova, Z., Wilen, C., Orchard, R., et al. (2016). Optimized sgRNA design to maximize activity and minimize off-target effects of CRISPR-Cas9. Nat. Biotechnol. *34*, 184–191.

Dudley, D.D., Chaudhuri, J., Bassing, C.H., and Alt, F.W. (2005). Mechanism and control of V(D)J recombination versus class switch recombination: similarities and differences. Adv. Immunol. *86*, 43–112.

Geisinger, J.M., Turan, S., Hernandez, S., Spector, L.P., and Calos, M.P. (2016). In vivo blunt-end cloning through CRISPR/Cas9-facilitated non-homologous end-joining. Nucleic Acids Res. *44*, e76.

Henser-Brownhill, T., Monserrat, J., and Scaffidi, P. (2017). Generation of an arrayed CRISPR-Cas9 library targeting epigenetic regulators: from high-content screens to in vivo assays. Epigenetics *12*, 1065–1075.

Horlbeck, M.A., Witkowsky, L.B., Guglielmi, B., Replogle, J.M., Gilbert, L.A., Villalta, J.E., Torigoe, S.E., Tjian, R., and Weissman, J.S. (2016). Nucleosomes impede Cas9 access to DNA in vivo and in vitro. eLife *5*, e12677.

Hsu, P.D., Lander, E.S., and Zhang, F. (2014). Development and applications of CRISPR-Cas9 for genome engineering. Cell *157*, 1262–1278.

Isaac, R.S., Jiang, F., Doudna, J.A., Lim, W.A., Narlikar, G.J., and Almeida, R. (2016). Nucleosome breathing and remodeling constrain CRISPR-Cas9 function. eLife *5*, 5.

Jensen, K.T., Fløe, L., Petersen, T.S., Huang, J., Xu, F., Bolund, L., Luo, Y., and Lin, L. (2017). Chromatin accessibility and guide sequence secondary structure affect CRISPR-Cas9 gene editing efficiency. FEBS Lett. *591*, 1892–1901.

Jinek, M., Chylinski, K., Fonfara, I., Hauer, M., Doudna, J.A., and Charpentier, E. (2012). A programmable dual-RNA-guided DNA endonuclease in adaptive bacterial immunity. Science *337*, 816–821.

Kalousi, A., and Soutoglou, E. (2016). Nuclear compartmentalization of DNA repair. Curr. Opin. Genet. Dev. *37*, 148–157.

Kosicki, M., Tomberg, K., and Bradley, A. (2018). Repair of double-strand breaks induced by CRISPR-Cas9 leads to large deletions and complex rearrangements. Nat. Biotechnol. *36*, 765–771.

Kundaje, A., Meuleman, W., Ernst, J., Bilenky, M., Yen, A., Heravi-Moussavi, A., Kheradpour, P., Zhang, Z., Wang, J., Ziller, M.J., et al.; Roadmap Epigenomics Consortium (2015). Integrative analysis of 111 reference human epigenomes. Nature *518*, 317–330.

Lemos, B.R., Kaplan, A.C., Bae, J.E., Ferrazzoli, A.E., Kuo, J., Anand, R.P., Waterman, D.P., and Haber, J.E. (2018). CRISPR/Cas9 cleavages in budding yeast reveal templated insertions and strand-specific insertion/deletion profiles. Proc. Natl. Acad. Sci. USA *115*, E2040–E2047.

Lindsay, H., Burger, A., Biyong, B., Felker, A., Hess, C., Zaugg, J., Chiavacci, E., Anders, C., Jinek, M., Mosimann, C., and Robinson, M.D. (2016). CrispRVariants charts the mutation spectrum of genome engineering experiments. Nat. Biotechnol. *34*, 701–702.

Maruyama, T., Dougan, S.K., Truttmann, M.C., Bilate, A.M., Ingram, J.R., and Ploegh, H.L. (2015). Increasing the efficiency of precise genome editing with CRISPR-Cas9 by inhibition of nonhomologous end joining. Nat. Biotechnol. *33*, 538–542.

Shen, M.W., Arbab, M., Hsu, J.Y., Worstell, D., Culbertson, S.J., Krabbe, O., Cassa, C.A., Liu, D.R., Gifford, D.K., and Sherwood, R.I. (2018). Predictable and precise template-free CRISPR editing of pathogenic variants. Nature. Published online November 7, 2018. https://doi.org/10.1038/s41586-018-0686-x.

Shou, J., Li, J., Liu, Y., and Wu, Q. (2018). Precise and predictable CRISPR chromosomal rearrangements reveal principles of Cas9-mediated nucleotide insertion. Mol. Cell *71*, 498–509.e4.

Taheri-Ghahfarokhi, A., Taylor, B.J.M., Nitsch, R., Lundin, A., Cavallo, A.-L., Madeyski-Bengtson, K., Karlsson, F., Clausen, M., Hicks, R., Mayr, L.M., et al. (2018). Decoding non-random mutational signatures at Cas9 targeted sites. Nucleic Acids Res. *46*, 8417–8434.

Tsai, S.Q., Zheng, Z., Nguyen, N.T., Liebers, M., Topkar, V.V., Thapar, V., Wyvekens, N., Khayter, C., Iafrate, A.J., Le, L.P., et al. (2015). GUIDE-seq enables genome-wide profiling of off-target cleavage by CRISPR-Cas nucleases. Nat. Biotechnol. *33*, 187–197.

Uusi-Mäkelä, M.I.E., Barker, H.R., Bäuerlein, C.A., Häkkinen, T., Nykter, M., and Rämet, M. (2018). Chromatin accessibility is associated with CRISPR-Cas9 efficiency in the zebrafish (Danio rerio). PLoS ONE *13*, e0196238.

van Heemst, D., Brugmans, L., Verkaik, N.S., and van Gent, D.C. (2004). End-joining of blunt DNA double-strand breaks in mammalian fibroblasts is precise and requires DNA-PK and XRCC4. DNA Repair (Amst.) *3*, 43–50.

van Overbeek, M., Capurso, D., Carter, M.M., Thompson, M.S., Frias, E., Russ, C., Reece-Hoyes, J.S., Nye, C., Gradia, S., Vidal, B., et al. (2016). DNA repair profiling reveals nonrandom outcomes at Cas9-mediated breaks. Mol. Cell *63*, 633–646.

Wang, T., Wei, J.J., Sabatini, D.M., and Lander, E.S. (2014). Genetic screens in human cells using the CRISPR-Cas9 system. Science *343*, 80–84.

STAR★METHODS

KEY RESOURCES TABLE

REAGENT or RESOURCE	SOURCE	IDENTIFIER
Antibodies		
Rabbit polyclonal anti-trimethyl-Histone H3 (Lys27)	Millipore	Cat# 07-449; RRID: AB_310624
Mouse monoclonal anti-Ezh2 (AC22)	Cell Signaling Technology	Cat# 3147; RRID: AB_2102420
Rabbit polyclonal anti-Histone H3 (acetyl K27)	Abcam	Cat# ab4729; RRID: AB_2118291
Rabbit polyclonal anti-mouse IgG H&L	Abcam	Cat# ab46540; RRID: AB_2614925
Mouse monoclonal anti-phospho-Histone H2A.X (Ser139)	Millipore	Cat# 05-636; RRID: AB_309864
HRP goat anti-rabbit IgG (Peroxidase)	Vector Laboratories	Cat# PI-1000; RRID: AB_2336198
Donkey polyclonal anti-mouse IgG AF488	Thermo Fisher	Cat# A-21202; RRID: AB_141607
Donkey polyclonal anti-mouse IgG AF568	Thermo Fisher	Cat# A10037; RRID: AB_2534013
Donkey polyclonal anti-mouse IgG AF647	Thermo Fisher	Cat# A-31571; RRID: AB_162542
Donkey polyclonal anti-rabbit IgG AF488	Thermo Fisher	Cat# A-21206; RRID: AB_2535792
Donkey polyclonal anti-rabbit IgG AF568	Thermo Fisher	Cat# A10042; RRID: AB_2534017
Chemicals, Peptides, and Recombinant Proteins		
Trichostatin A	Sigma	Cat# T1952
GSK126 (EZH2 inhibitor)	Cayman Chemical	Cat# 15415
Critical Commercial Assays		
MiSeq Reagent Kit v3	Illumina	Cat# MS-102-3003
DNeasy Blood & Tissue Kit	QIAGEN	Cat# 69506
SureSelectXT Custom 0.5-2.9Mb library	Agilent	Cat# 5190-4816
QIAquick Gel Extraction Kit	QIAGEN	Cat# 28706
QIAquick PCR Purification Kit	QIAGEN	Cat# 28106
Herculase II Fusion DNA polymerase	Agilent	Cat# 600675
CellTiter 96 Aqueous One Solution	Promega	Cat# G3582
Deposited Data		
Targeted DNA-seq of Human HepG2 cells following editing with CRISPR/Cas9	EBI ArrayExpress	ArrayExpress: E-MTAB-7095
Targeted DNA-seq of Human HepG2 cells following editing with CRISPR/Cas9 upon chromatin modulation with TSA and EZH2i	EBI ArrayExpress	ArrayExpress: E-MTAB-7091
Experimental Models: Cell Lines		
Human: HepG2 cells	The Francis Crick Cell Services Department	N/A
Human: HEK293-T cells	The Francis Crick Cell Services Department	N/A
Oligonucleotides		
Primers used in this study (see Table S7)	This paper	N/A
Recombinant DNA		
pLenti_BSD_sgRNA	Henser-Brownhill et al., 2017	N/A
Software and Algorithms		
FastQC	https://www.bioinformatics.babraham.ac.uk/projects/fastqc/	N/A
BBMap 36.59	https://sourceforge.net/projects/bbmap/	N/A
R 3.3.2 - 3.4.4	The R Project for Statistical Computing	https://www.r-project.org/

(*Continued on next page*)

Continued

REAGENT or RESOURCE	SOURCE	IDENTIFIER
CrispRVariants	https://github.com/HLindsay/CrispRVariants	N/A
Python 3.7	Python Software Foundation	https://www.python.org/
Apache MXNet (v1.2.0) (python 3 API)	The Apache Software Foundation	https://mxnet.apache.org/
Custom analysis scripts	This paper	https://github.com/luslab/crispr-indels
Other		
van Overbeek et al., 2016	Sequence Read Archive	SRP076796
HepG2 H3K9ac, H3K27ac ChIP-seq and DNase-seq	Kundaje et al., 2015	http://www.roadmapepigenomics.org/
HEK293 K3K9ac ChIP-seq	Cistrome DB	58997
HEK293 K3K27ac ChIP-seq	Cistrome DB	43073
HEK293 DNase-seq	Gene Expression Omnibus	GSM1635901-6
K562 K3K9ac ChIP-seq	Cistrome DB	45406
K562 K3K27ac ChIP-seq	Cistrome DB	55731
K562 DNase-seq	Cistrome DB	45020 & 45021

CONTACT FOR REAGENT AND RESOURCE SHARING

Further information and requests for resources and reagents should be directed to and will be fulfilled by the Lead Contact, Paola Scaffidi (paola.scaffidi@crick.ac.uk).

EXPERIMENTAL MODEL AND SUBJECT DETAILS

Cell lines

HepG2 cells, of male origin, were cultured in Minimum Essential Media (MEM) with 10% FBS, and HEK-293T cells, of likely female origin, were cultured in Dulbecco's Modified Eagle's Medium (DMEM) with 10% FBS. All media was supplemented with 2mM L-glutamine, 100U/mL penicillin, and 100 μg/mL streptomycin. All cell lines were maintained at 37°C and 5% CO_2. Cas9-expressing HepG2 cells were generated as previously described (Henser-Brownhill et al., 2017). For all experiments, Cas9 expression was induced with 1 μg/mL doxycycline 1 day prior to infection with the sgRNAs and sustained until cells were harvested for genomic DNA extraction (QIAGEN). All cell lines were obtained from the Francis Crick Institute Cell Services Department and have been STR authenticated and tested negative for mycoplasma.

METHOD DETAILS

sgRNAs pool generation

sgRNA pools were generated by combining equal volumes of saturated bacterial culture from the arrayed library described in Henser-Brownhill et al. (2017), and extracting the resulting plasmid libraries. Six different pools were generated and independently transduced into HepG2 Cas9-expressing cells. This was necessary to reduce the library complexity and allow efficient detection of indels despite the high number of unedited sequences in the cell population – each sgRNA only infected a limited number of cells. We first tested three pools targeting 100 sites each (pools 100_1, 100_2 and 100_3). Once we confirmed efficient indel detection, we generated three sgRNA pools targeting 450 sites each (pools 450_5, 450_6, 450_7). 450 pools contained three distinct sgRNAs targeting the same 450 genes. 100 pools mainly contained sgRNAs present in the 450 pools with a few additional ones (Table S1). Although pools were transduced and processed independently, indel analysis was performed integrating data from the different pools. When assessing efficiency of indel formation, only data from 450 pools were used. This was done because indel counts for sgRNAs present in both 450 and 100 pools were artificially higher than those detected at sites targeted only with the 450 pools. When assessing editing precision, data from both 100 and 450 pools was combined, as frequencies of individual indels are not affected by differences in indel counts.

Viral transductions

Transduction of sgRNAs was performed using high titer virus, at an estimated MOI of at least 10, to increase the fraction of edited cells in the population for each sgRNA. To produce virus, 80% confluent HEK293T cells were transfected with the sgRNA pools (pLenti_BSD_sgRNA plasmids), packaging plasmids (psPax2 and pMD2G) and pAdVantage at a ratio of 3:1 DNA to FugeneHD (Promega). 24h after transfection viral particles were collected, filtered through a 0.45 μm filter and used to infect Cas9-expressing HepG2 cells in the presence of 5μg/ml Polybrene (Santa Cruz). To increase infection efficiency, plated cells were spun for 2h at

2000rpm soon after the virus-containing supernatant was added. A second infection was carried out using viral particles collected 48h after transfection. Cells were not spun for the second infection. Transduced cells were selected with 4 μg/mL blasticidin (Merck), starting 24h after the first infection, and genomic DNA was extracted 5 days after infection (QIAGEN).

Timing of CRISPR-mediated editing

In order to experimentally determine the kinetics of indel formation, sgRNAs targeting 3 sites (ACTL6A.5, ASF1B.7 and SMARCD2.1) were individually transduced into Cas9-expressing HepG2 cells, using high titer virus to ensure efficient infection of all cells. Genomic DNA was isolated from infected cells (QIAGEN) for 5 consecutive days and editing of the target sites quantified by Sanger sequencing (Herculase II Fusion, Agilent) and TIDE analysis (https://tide.deskgen.com/) (See Table S7 for primers). To confirm the absence of possible phenotypic consequences induced by gene knock-out after 5 days, which may confound the results, cells infected with an EZH2-targeting sgRNA were analyzed by immunofluorescence to quantify the levels of both EZH2 and its associated mark H3K27me3. Based on these experiments, 5 days post-infection was concluded to be the optimal length for performing all subsequent experiments.

Protein detection

Western blot analysis and immunofluorescence microscopy were performed using anti-H3K27ac (Abcam ab4729), anti-H3K27me3 (Millipore 07-449), anti-ɣH2A.X (Millipore 05-636), anti-EZH2 (CST 3147) and Alexa Fluor- or HRP-conjugated secondary antibodies following standard protocols.

Chromatin modulation and ChIP-qPCR

To investigate the effect of chromatin on CRISPR-mediated DNA editing, HepG2 cells were treated with the HDAC inhibitor Trichostatin A (Sigma), which induces histone hyperacetylation, and the EZH2 inhibitor GSK126 (Cayman Chemical), which globally reduces H3K27me3 levels. Cells pre-treated with TSA (11nM or 100nM) or GSK126 (0.3μM and 3μM) for 5 days were infected with sgRNAs targeting the ACTL6A.5, ASF1B.7, BRD2.7, MBD3L1.6, MSH6.2 and SMARCD2.1 sites. Treatment was continued for an additional 5 days while indels were induced. Compounds were refreshed daily over the course of the experiment. Successful alteration of histone acetylation at the target sites was confirmed by ChIP-qPCR of H3K27ac in cells either untreated (NT) or treated with TSA (100nM). For both conditions, 8 million HepG2 cells were fixed with 1% formaldehyde for 10 min at room temperature, treated with 125mM glycine for 5min at RT, washed three times with ice-cold PBS and scraped off cell culture plates in PBS supplemented with 10% FBS. Cell pellets were resuspended in 0.6mL of IP buffer (1:1 of SDS buffer (0.5% SDS, 0.2% NaN_3, 5mM EDTA pH 8, 50mM TRIS pH 8, 100mM NaCl): Triton buffer (5% Triton X, 0.2% NaN_3, 5mM EDTA pH 8, 100mM NaCl, 100mM TRIS pH 8)) supplemented with protease inhibitors (1:100, Cell Signaling Technology) and incubated for 15 min on ice. Chromatin was subsequently sheared to 200-500bp with 2 cycles of 30sec ON/OFF using the Bioruptor sonicator (Diagenode). Chromatin from each biological replicate was divided into 2 and 200μg of sample were incubated overnight at 4°C with 8μg of either anti-acetyl H3K27 (Abcam ab4729) or control anti-rabbit IgG (Abcam ab46540). In all cases, 10% of each sample was kept as input. Next, 30μL of Pierce Protein G magnetic beads (Invitrogen) were added per sample and incubated an additional 4h at 4°C. All samples were then washed 3x with low salt wash buffer (1% Triton X, 0.1% SDS, 2mM EDTA pH 8, 20mM TRIS pH 8, 150mM NaCl) and 1x with high salt wash buffer (1% Triton X, 0.1% SDS, 2mM EDTA pH 8, 20mM TRIS pH 8, 500mM NaCl) with the use of a magnetic rack. Subsequently, 120μL of decrosslinking buffer (1% SDS, 100mM $NaHC0_3$) was added to all samples and inputs and incubated overnight at 65°C. All decrosslinked samples were purified using the QIAquick PCR purification kit (QIAGEN) and eluted in 45μL of Nuclease-free water. ChIP samples were analyzed on a CFX96 real-time PCR detection system (Bio-rad) using SsoAdvanced Universal SYBR Green Supermix (Bio-rad). All samples were run in triplicates and normalized to the 10% input that was retained before pulldown.

Cell proliferation

To examine the effect of the chromatin-modulating compounds on HepG2 cell proliferation, 8,000 HepG2 cells were plated per well of a 96-well plate and treated with TSA (11nM or 100nM) or GSK126 (0.3μM or 3μM) for 5 days. On a daily basis, 20μL of Cell-Titer 96 Aqueous One Solution (Promega) were added per well in triplicates and following incubation at 37°C for 1h, the Optical Density of each well was read at 490nm as a measure of the number of cells per well. The growth rate of the cells was normalized to the number of cells on day 1.

Library preparation and deep sequencing

sgRNA representation in pools

To assess the representation of individual sgRNAs in the plasmid library, amplicons containing the sgRNA sequences were generated as previously described (Henser-Brownhill et al., 2017). Briefly, PCR amplicons containing the P5 and P7 Illumina adaptors were generated using the high-fidelity Herculase II polymerase kit (Agilent), and the resulting products extracted from an agarose gel (QIAGEN). Purified products were sequenced with either a HiSeq 2500 or a MiSeq using custom sequencing and indexing primers (SeqP and IndexP, Table S7). Following sample demultiplexing, all sgRNA sequences were trimmed and aligned to the target sequences to assess sgRNA representation (normalized read count).

Large scale indel sequencing

To identify CRISPR-mediated editing at targeted regions, DNA libraries enriched for the targeted sites were generated using the SureSelect Target enrichment kit (Agilent) following the manufacturer's instructions. Capture probes were designed to cover 2Kb regions centered on each target site. When multiple target sites were located in the same exon, the 2Kb region was centered on the exon middle point. Probe tiling parameters were: Tiling density: 1x; Masking: Least Stringent; Boosting: Maximize Performance. All samples were sequenced using Paired End 100bp runs on a HiSeq 4000 sequencer, multiplexing 2 samples per lane. Approximately 200 million reads were obtained for each sample. Analysis of sequenced regions confirmed good enrichment of the targeted regions (Figure S2A).

Small scale indel sequencing

For validation experiments and experiments assessing the effect of chromatin modulation, indels induced at 6 selected sites were examined. In these experiments, a two-step PCR was performed on biological duplicates to generate a library of PCR amplicons. For the first PCR, 150ng of the corresponding gDNA were amplified for 20-22 cycles using the Herculase II polymerase kit yielding products of ~500bp (See Table S7 for primers). Next, PCR products were purified as per manufacturer's instructions (QIAGEN) and 1μl of the resulting product was used as a template for the second nested PCR reaction in which primers containing barcodes and adapters for the sequencing reaction were added. Overall, a library of 60 individually barcoded amplicons of ~300bp was generated (See Table S7 for primers). Samples were purified in a 96-well format (Zymo Research) and sequenced on a 300bp paired-end run on a MiSeq using standard Illumina sequencing primers (See Table S7 for primers). The long 300bp reads allowed assessment of both long and short indels.

Sequencing read alignment

The quality of the sequenced reads was assured using FastQC. For alignment, we used BBMap (v. 36.59) as it is a global aligner that is able to align longer indels. Alignment was carried out against the UCSC hg19/GRCh37 genome assembly.

Indel identification

Large scale indel sequencing

In order to robustly identify the reads that contained indels we adopted a two-stage alignment strategy. In the first phase we aligned the reads to the genome disallowing any reads that contained indels. We discarded reads that aligned in a proper pair in this phase and took the remainder forward. In the second phase we aligned the remaining reads to the genome, this time setting a soft threshold allowing indels up to 2000bp. Duplicates were marked using Picard (v. 2.1.0). Reads that were marked as duplicates, or that had a mapping quality score of less than 38 were filtered using samtools (v. 1.2) and sambamba (v. 0.6.0). This two-phase approach was necessary to delineate, for a given target amplicon, between reads from cells uninfected with the corresponding sgRNA and reads from cells with successful transfections, on account of the pooling of sgRNAs. For a given amplicon, only a small proportion of the total number of cells would have been transfected with the sgRNA targeting the site contained within it. We know that aligned reads containing indels arise from appropriately transfected cells. However, our approach forces the aligner to choose an alignment with no indels over one with indels for the multiple possibilities for a given read. With this approach we can improve our confidence that the reads with indels are not background noise or alignment errors. Because of the experimental approach, the sensitivity of our method is inherently limited, and it is likely that indels occurring at low frequency are not detected. Furthermore, kinetically slow repair events may be underrepresented in our dataset. Nevertheless, the observation that most targets are identified as imprecise or middle indicates that there is no significant bias toward most frequent indels. Furthermore, complementary studies using alternative experimental approaches (Shen et al., 2018) observed a very similar distribution of precision groups, confirming the reliability of our method.

Indel identification was performed in R (v. 3.4.4) using custom scripts. The location and size of indels in reads were identified from the CIGAR string. Indels were only considered valid if they occurred within 5 nucleotides of the Cas9 cleavage site (defined as 6 nucleotides upstream of the end of the guide RNA including the PAM sequence). Any indels that could also be detected in the control HepG2 sample were removed as probable somatic mutations in this cancer cell line. To ensure robust estimate of indel frequencies, we filtered out target sites that had a low overall indel count (indels identified in fewer than 10 reads in total across all samples and replicates, where present).

Assessment of indel identification approach

To assess possible confounding effects from sequencing errors, particularly given the depth of sequencing, we performed two complementary analyses. First, we assessed the number of indels detected at each target site (within 5 nucleotides of the Cas9 cleavage site) in the wild-type sample without Cas9 induction and sgRNA transduction (without filtering for probable HepG2 somatic mutations). Second, we leveraged the fact that all targeted regions in the whole library were pulled down and sequenced to a similar depth in all experiments, irrespective of whether they were targeted in that particular pool or not. Therefore we compared the number of indels in both replicates from the 450 pool experiments at each target site in the experiment where the corresponding sgRNA was in the transfected pool, with the mean of the number of indels in both replicates from the two other 450 pool experiments where the corresponding sgRNA was not. This provided an estimate of the occurrence of sequencing errors in our experimental setup within 5 nucleotides of the Cas9 cleavage site.

Small scale indel sequencing

Before alignment, paired end reads were merged using BBMerge (v. 36.59). After alignment, duplicates were marked using Picard (v. 2.1.0). Reads that were marked as duplicates, or that had a mapping quality score of less than 38 were filtered using samtools (v. 1.2) and sambamba (v. 0.6.0). The R package CrispRVariants (Lindsay et al., 2016) was used to identify indels.

Characterization of target sites

Throughout, we used all detected indels from both 100 and 450 pools to characterize the targets, except when assessing for efficiency where indels from the 450 pools only were used to ensure an unbiased analysis of each target site as explained above.

Frameshifts and indel size

Indels were assessed for their frameshift potential by the divisibility of their size by 3. To identify patterns in the indel size profiles at target sites, we calculated the frequency of each size of indel (considered in bins of insertions greater than 1 nucleotide, insertions of 1, and deletions of 1, 2, 3, 4, 5, 6, 7, 8, 9, 10 and greater than 10). We performed unsupervised hierarchical clustering using the Ward D2 method to categorize groups of target sites based on their indel size profiles.

Precision

We also categorized target sites by calculating the frequency of each distinct indel at each target site. The most frequent indel was termed the commonest; ties were broken by prioritizing insertions over deletions, and then by longest deletion. The precision of indel generation at a target site was defined based on the frequency of the commonest indel: imprecise $\leq$ 0.25, 0.25 < middle $\leq$ 0.5, precise > 0.5.

Sequence homology

The presence of MH of n nucleotides was assessed in the deletions. The 5′ n nucleotides of the deleted sequence were compared with the first n nucleotides downstream of the 3′ join. Likewise, the 3′ n nucleotides of the deleted sequence were compared with the last n nucleotides upstream of the 5′ join. If there was a match, this was considered as MH. For each deletion sequence, values of n ranging from 1 to 50 (or the length of the deletion, whichever was shortest) were used. The largest matching n was considered the size of the MH.

Insertion homology was assessed by extracting the inserted nucleotide from the read sequence using the CIGAR string. This was compared with the nucleotide in the -4 position of the protospacer to assess for matches. When assessing the commonest insertion, we only considered target sites that had 5 or more insertions. Where the inserted nucleotide either creates, or lies within a short repetitive stretch; e.g., "A" inserted adjacent to "A" creating "AA," or "T" inserted adjacent to/within "TT" creating "TTT"; it is not possible to identify precisely which of these nucleotides is the inserted position. The aligner arbitrarily assigns the first position to the inserted nucleotide.

Analysis of van Overbeek data

For the van Overbeek 'spacer' target sites, aligned BAM files were obtained from the Sequence Read Archive for all time points in HCT116, HepG2 and K562 cell lines. Indel identification was performed in R (v. 3.4.4) using custom scripts. The location and size of indels in reads were identified from the CIGAR string. Indels were only considered valid if they occurred within 5 nucleotides of the Cas9 cleavage site (defined as 6 nucleotides upstream of the end of the guide RNA including the PAM sequence). For a given time point and cell type, indels that occurred with < 1% frequency were filtered, as were sites that had < 10% editing efficiency. Downstream analyses were performed as detailed in 'Characterization of target sites' above.

Indel profiles upon chromatin modulation

Mutation efficiency was assessed using the mutationEfficiency function from CrispRVariants (Lindsay et al., 2016), considering single nucleotide variants as non-variants. To compare the counts of indels across the different conditions, in order to assess the contribution of each indel to the changes in efficiency, the raw counts for each indel in each condition were normalized to the library size for that condition. Indels that constituted less than 1% of the library size in any condition were filtered out.

To assess the effects of chromatin modulation on the indel profile of target, over and above the effects on efficiency, we performed a different normalization on the raw counts. We divided by a size factor (the total number of indels detected in a condition). In this way, we could compare the relative contribution of each indel to the overall indel profile across the different conditions. After normalization, only the most frequent 10 indels in the untreated condition were used.

Analysis of chromatin environment

DNase-seq and H3K9ac and H3K27ac ChIP-seq fold-enrichment data for in HepG2 cells were obtained pre-processed from the Roadmap Epigenomics consortium (Kundaje et al., 2015). We calculated the mean fold-enrichment signal in a 500bp window centered on the cleavage site of the six validation targets. For the van Overbeek 'spacer' target sites, preprocessed coverage files were obtained for DNase-seq, H3K9ac and H3K27ac ChIP-seq for HEK293 and K562 cell lines aligned to GRCh38 from sources indicated in the Key Resources table. Data quality was assessed using Cistrome's tools and manual inspection. 500bp windows centered on the cleavage site were created and converted from GRCh37 to GRCh38 using the UCSC liftOver tool. The signal in each window was extracted using Deeptools. For visualization, the mean signal for each dataset was centered and scaled across all the target sites.

Analysis of nucleotide influence

Artificial neural network

To estimate editing precision, we designed an artificial neural network (ANN) that uses the raw sgRNA sequences as input: 20 individual nucleotides, plus the PAM sequence (as a rudimentary internal control). All variable nucleotides were encoded using one hot encoding. The input layer of the network therefore has 86 nodes, with each of the 21 variable nucleotide positions in the 23nt sgRNA target sequence represented by 4 binary inputs, and the 2 constant 'G's in the PAM sequence represented as single constant values. These are followed by a single hidden layer containing 512 neurons using rectified linear unit (ReLU) activation functions, connected to a single output node, followed by a softplus activation function. Our loss function was mean square error (L2 norm loss). The model parameters were initialized using Xavier initialization. In summary, the weights were initially filled with random numbers [-*c*, *c*] where:

$$C = \sqrt{\frac{2.24}{0.5 \times (n_{in} + n_{out})}}$$

Here, n_{in} is the number of neurons preceding weights and n_{out} is the number of neurons proceeding weights. 80% (n = 519) of our sgRNAs were randomly selected for use as a training set, with 20% (n = 130) held out as a test set. To ensure consistency and to mitigate bias introduced by particular sets of sgRNAs in the training set, we validated our model by performing bootstrapping with replacement (taking a random sample of 80% (n = 415) of our training sgRNAs each time) before training the final model (final validation RMSE = 0.15 ± 0.003). The final ANN was trained for 800 epochs using stochastic gradient descent with Nesterov momentum set to 0.9, a learning rate of 0.001, and a batch size of 100. The final model's RMSE was 0.14 for the train set, 0.18 for the test set, and 0.16 for van Overbeek et al. To identify key sequence positions with the greatest influence on editing precision, we conducted a permutation nucleotide importance analysis by systematically randomizing each nucleotide in the test set at the respective position. We maintained the original prior-distribution by shuffling the column values before one hot encoding. The mean decrease in accuracy was recorded as the reduction in R^2 from predictions made with the unaltered sequences. We also recorded the difference in predictive statistical significance (Wald test p values). We performed the nucleotide randomization 10 times and report the average percentage reduction in R^2 for neutralized positions. We considered an average increase in Wald test p values to > 0.05 as having abolished the predictive significance of the model. The ANN was built, trained, and deployed using *Apache MXNET* (python 3 API) v. 1.2.0.

LASSO multi-regression model

To corroborate the results of our non-linear ANN model, and obtain the coefficients of the most important linear correlations with observed indel frequencies, we constructed a linear model optimized for generalization using L_1 regularization by deploying a least absolute shrinkage and selection operator (LASSO) algorithm. Here the aim is to minimize the objective function:

$$\frac{1}{2n_{samples}} \| Xw - y \|_2^2 + \alpha \| w \|_1$$

Where regularization parameter α is a constant and $\|w\|_1$ is the L_1 regularized parameter coefficient vector. Our training set was 80% of our data (n = 519) selected at random, with 20% (n = 130) held out to test the model. The coefficients were fitted using coordinate descent and the regularization parameter α (0.002592943797404667) selected by 10-fold cross validation on the training set. The final model's RMSE was 0.15 for the train set, 0.17 for the test set, and 0.15 for van Overbeek et al. The LASSO was built, trained, and deployed using *scikit-learn v. 0.19.1* for python 3.

QUANTIFICATION AND STATISTICAL ANALYSIS

Non-parametric statistical tests were used as appropriate and p-values were adjusted for multiple testing where necessary. Each specific test is indicated in the main text or figure legend, as well as the exact value of N and what N represents. In boxplots, the bottom and top of boxes indicate the 25th and 75th percentiles, respectively, and middle lines indicate medians. Whiskers indicate the lowest and highest data points within 1.5 × interquartile range from the box. A significance level of 0.05 was used throughout.

DATA AND SOFTWARE AVAILABILITY

The accession numbers for the sequencing data generated in this study are EBI ArrayExpress: E-MTAB-7091, E-MTAB-7095. Custom scripts are available at https://github.com/luslab/crispr-indels.

Molecular Cell, Volume *73*

Supplemental Information

Target-Specific Precision

of CRISPR-Mediated Genome Editing

Anob M. Chakrabarti, Tristan Henser-Brownhill, Josep Monserrat, Anna R. Poetsch, Nicholas M. Luscombe, and Paola Scaffidi

Figure S1. Related to Figure 1

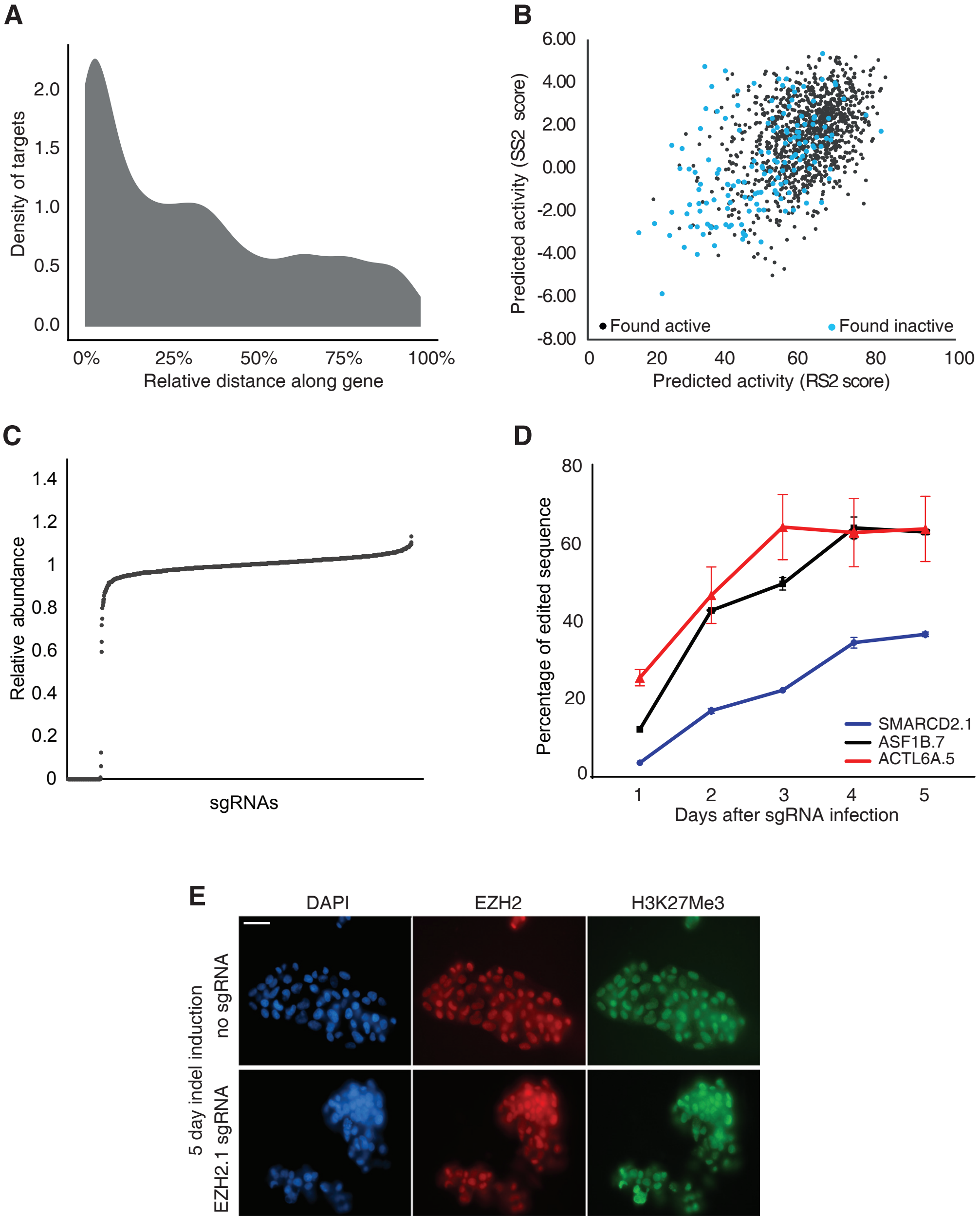

Figure S1. Characteristics of the sgRNA pools. Related to Figure 1.

A. Distribution of cleavage site locations of all target sites along the gene body.

B. Correlation between the indicated scores of predicted sgRNA activity for the used guides obtained from two distinct algorithms (Chari et al., 2017; Doench et al., 2016). sgRNAs that induced detectable indels are shown in black, while inactive sgRNAs are in blue. Note that several sgRNAs with high predicted activity were found to be inactive. Only sgRNAs with counts > 0 in C are shown.

C. Relative abundance of each sgRNA in the 450 pools. Values represent sequencing reads normalized to the median value of the three pools combined. With the exception of a few undetectable sgRNAs, all guides are homogeneously represented in the pools.

D. Time course of CRISPR-mediated editing at the indicated sites, after infection of individual sgRNAs. Values are mean ± standard deviation from two technical replicates. The percentage of edited sequence was estimated by TIDE analysis (Brinkman et al., 2014).

E. Immunofluorescence microscopy of the indicated samples using anti-EZH2 (red) and anti-H3K27me3 (green) antibodies, showing no detectable reduction in EZH2 or histone modification levels 5 days after indel induction. Nuclei are counterstained with DAPI (blue). Scale Bar 20 µm. EZH2.1 sgRNA had been individually transduced in Cas9-expressing cells using the same conditions used for the pooled sgRNAs, serving as a reporter of indel formation efficiency in the large-scale experiment. TIDE analysis of genomic DNA showed an editing efficiency of 37.6% at the EZH2.1 site.

Figure S2. Related to Figure 1

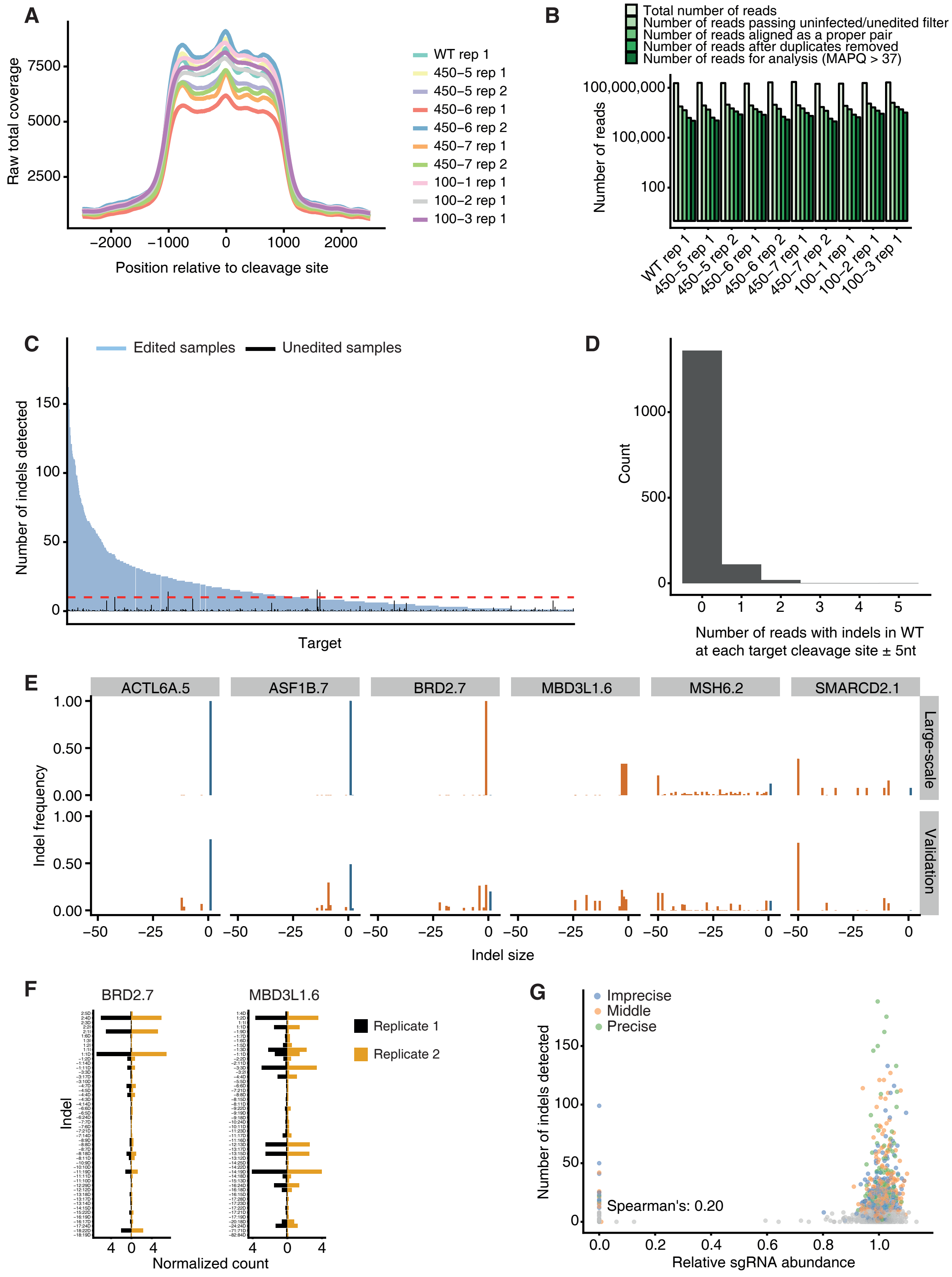

Figure S2. Indel detection metrics and validation of indel profiles. Related to Figure 1.

A. Raw total read coverage for each experiment over the region around the cleavage site that was selectively isolated by target capture.

B. Alignment metrics for the large-scale experiments. For each experiment, the number of reads at each stage of processing is shown. The filtering strategy used to detect indels robustly is described in the Methods section.

C. Number of total indels detected at each target site when using the 450 sgRNA pools (summed across both biological replicates). The number of indels detected in unedited control samples (see methods) is shown in black. The dashed line is at 10, reflecting the threshold that was set for the downstream analysis.

D. Number of indels detected at target sites in the wild-type experiment without Cas9 induction; data from one biological replicate.

E. Comparison of indels detected at 6 target sites in the large-scale experiment and the validation experiment.

F. Indel profiles for two biological replicates at the indicated target sites probed by high-coverage sequencing in validation experiments. Indel nomenclature: is [start coordinate relative to cleavage site]:[size][insertion or deletion]. Counts are normalized to the total library size for each experiment.

G. Relationship between the number of total indels detected at each target site and the abundance of the associated sgRNA in the pools. Note that some sgRNAs that were undetectable in the pools by next-generation sequencing induced indels at their target sites. Presence of the undetected, indel-inducing sgRNAs was confirmed by Sanger sequencing of the individual guides in the original arrayed library. These “undetectable” sgRNAs are included in C. sgRNAs are color-coded based on the groups described in Fig. 3A. Grey targets are those excluded from the downstream analysis as they induced less than 10 indels.

Figure S3. Related to Figures 2 & 3

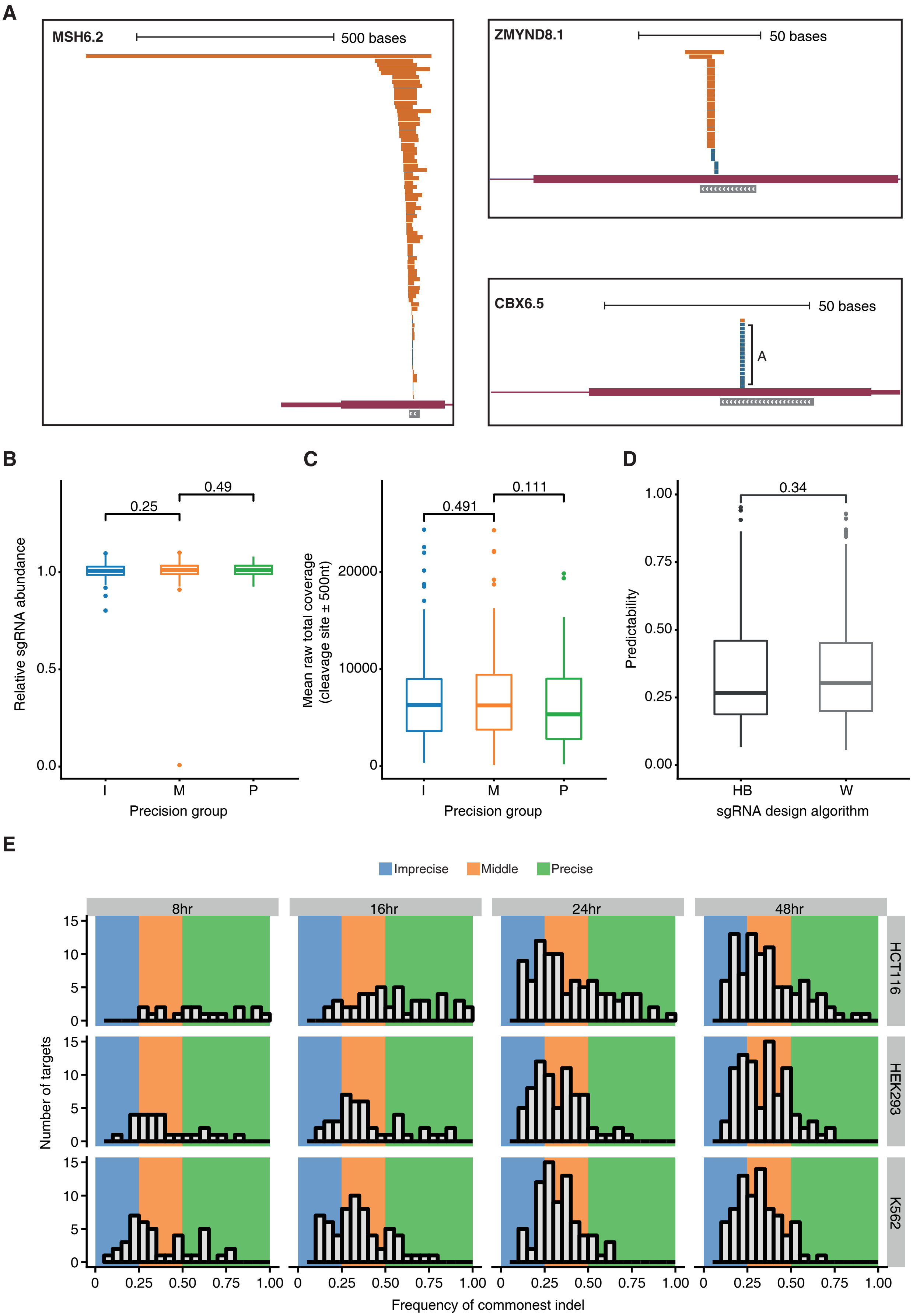

Figure S3. Editing precision groups of targets. Related to Figures 2 & 3.

A. Example sites showing a wide range of distinct indels (MSH6.2) or strong preference for a specific deletion (ZMYND8.1) or insertion (CBX6.5). Deletions are shown in orange, insertions in blue, with the gene body colored in plum and the sgRNA binding position in grey. Chevrons indicate strand.

B-C. Relationship between sgRNA abundance (B) and raw total read coverage (C) and assignment of precision group to target sites. Statistical testing: Kruskal-Wallis test followed by Dunn's test for multiple comparisons with Benjamini-Hochberg correction for multiple testing.

D. Relationship between the sgRNA design algorithm and assignment of precision group to target sites. HB and W indicate Henser-Brownhill design (purely based on sgRNA specificity) and Wang design (optimized for activity), respectively (Henser-Brownhill et al., 2017). Statistical testing: Kruskal-Wallis test.

E. Distribution of commonest indel frequencies at target sites from the van Overbeek dataset (van Overbeek et al., 2016) at the indicated times after sgRNA nucleofection. The background indicates three groups of sites as defined based on their editing precision. Note that a stable distribution is observed by 24h. Targets with an editing efficiency of < 10% at a time-point have been filtered out. The 4h time point has been omitted as no sites passed the threshold for HEK293.

Figure S4. Related to Figures 5

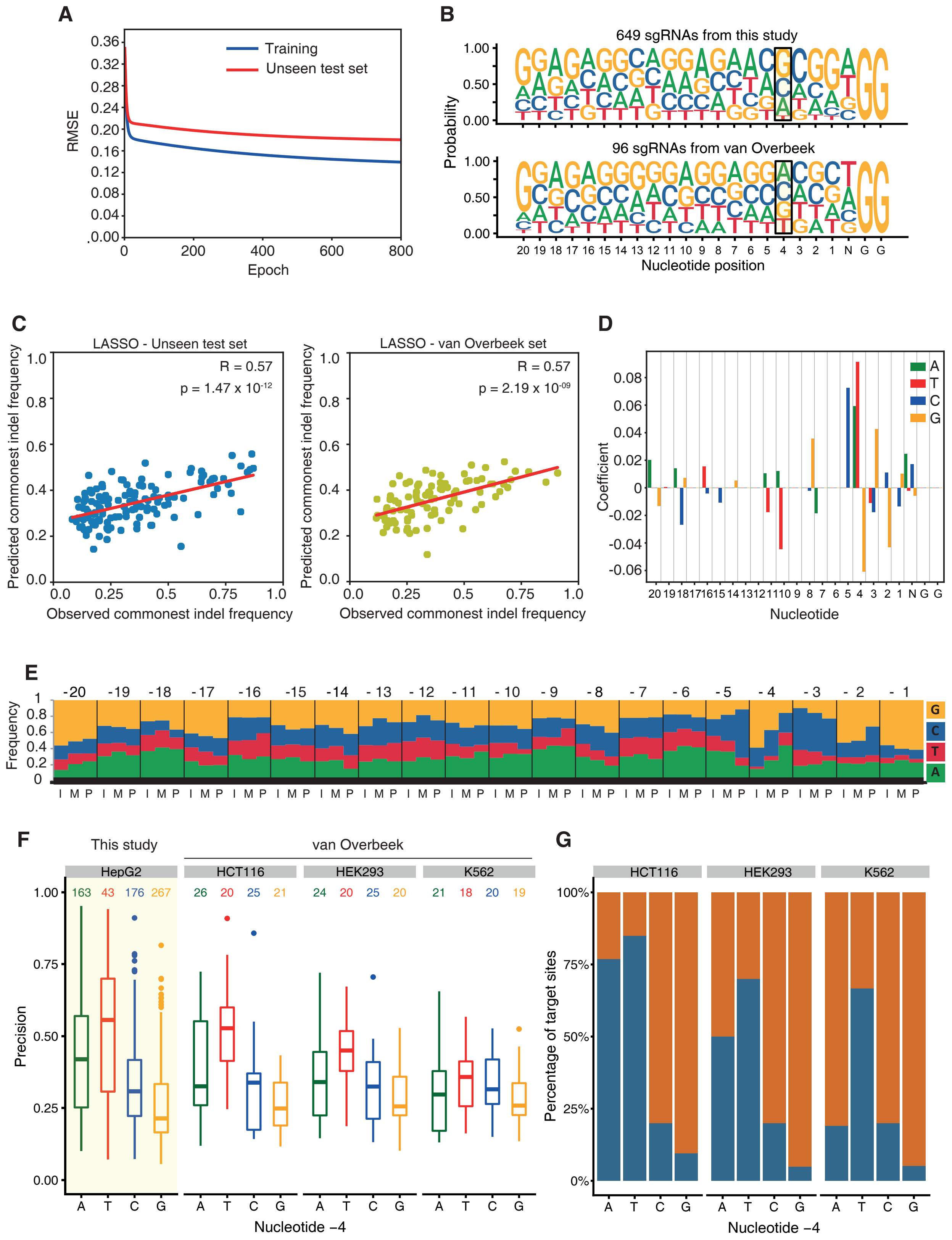

Figure S4. Robust predictability of editing outcome based on protospacer nucleotide composition. Related to Figure 5.

A. Final model training for the artificial neural network, with corresponding test error. Epoch indicates the number of iterations the network was presented with the training dataset.

B. Sequence logo showing the distribution of nucleotides at each position of the sgRNAs used here (above) and used by van Overbeek *et al.* (below).

C. Correlation between the observed precision at a given target site and that estimated by the LASSO model, using our test set (left) and the van Overbeek set (right). The best model selected after 10-fold cross-validation is shown (see Methods). R: correlation coefficient. Statistical significance testing: Wald χ^2 test.

D. Contribution of each nucleotide of the protospacer to editing precision as estimated by the LASSO model. Positive and negative values indicate that the nucleotide favor or disfavor precision, respectively.

E. Frequencies of each nucleotide in the protospacer sequence for each precision group. I: imprecise, M: middle, P: precise.

F. Precision of targets with the indicated nucleotide at position -4 in the sites targeted here (HepG2, shaded in yellow) compared with that calculated for the 96 van Overbeek *et al.* target sites in 3 different cell lines at the 48h time point.

G. Percentage of target sites with a preference (commonest indel) for insertions or deletions based on the nucleotide at position -4 calculated for the 96 van Overbeek *et al.* target sites in 3 different cell lines.

Figure S5. Related to Figures 6 & 7

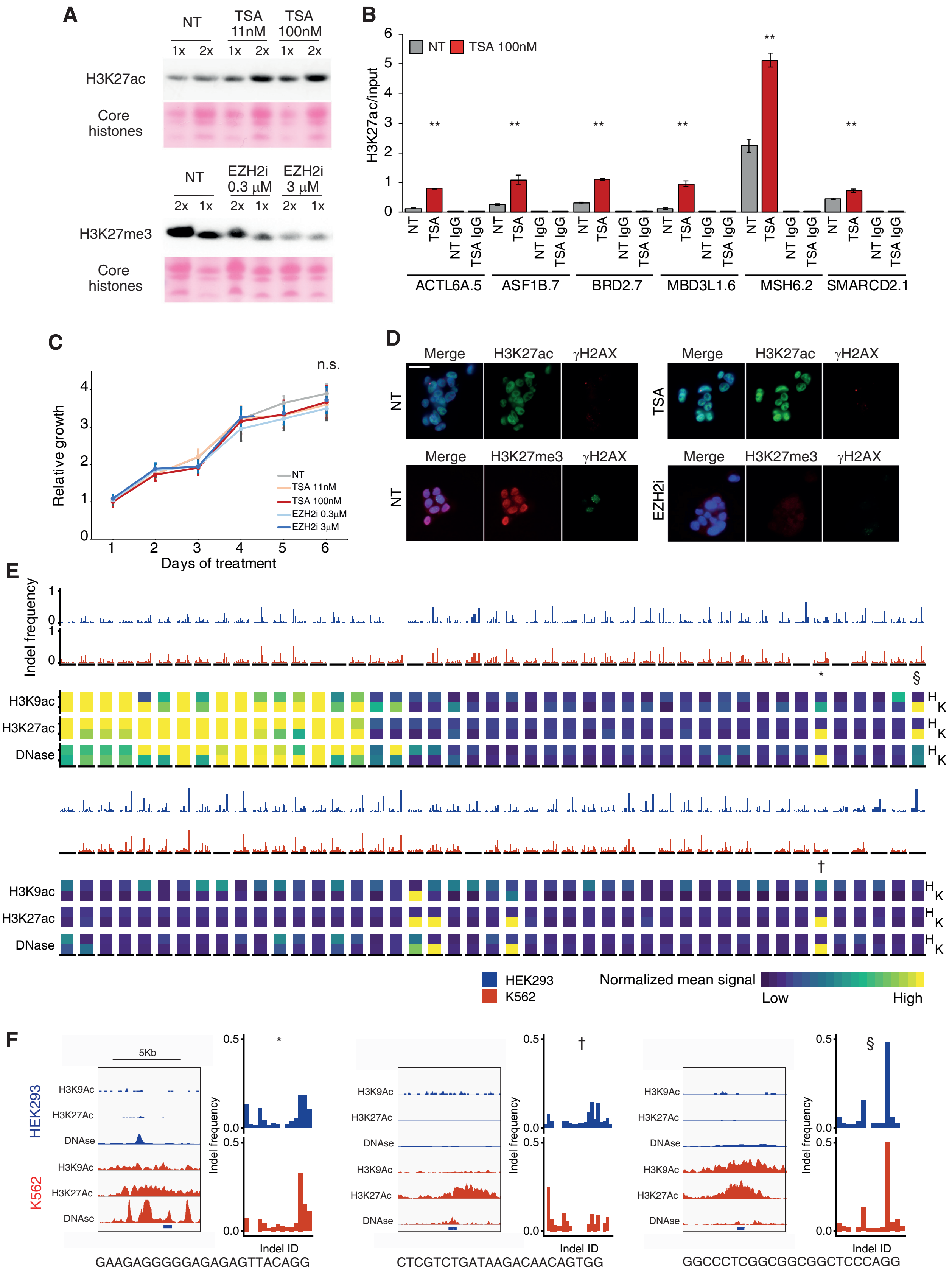

Figure S5. Chromatin modulation by TSA and EZH2i treatment. Related to Figures 6 & 7.

A. Western blot analysis of HepG2 cells untreated (NT) or treated with the indicated compounds. A dose-dependent increase in H3K27ac is observed in response to TSA (above), while EZH2i induces a dose-dependent reduction in H3K27me3 (below). Ponceau S staining of the core histones is shown as loading control. Two different amounts of protein lysate (1x and 2x) are loaded for each condition to allow a more quantitative assessment of the differences.

B. ChIP-qPCR showing increase of H3K27ac upon TSA treatment at the indicated sites. Values are mean ± standard deviation from two independent experiments. Values of pulled-down chromatin are normalized to 10% input chromatin. Statistical testing: Mann Whitney test (p value of < 0.01 shown as two asterisks). Signal for H3K27ac was enriched compared to IgGs for all sites in untreated (NT) cells. Similar experiments were performed upon EZH2i treatment, but due to intrinsically low levels of this mark in HepG2 cells we were unable to detect H3K27me3 at the sites. Even positive control genes (e.g. WT1 or HOXB9 promoter) showed minimal enrichment over the IgG control, while they showed strong enrichment in human fibroblasts used as a control cell line (not shown).

C. Proliferation curves of HepG2 cells treated with the indicated compounds. Values are mean ± standard deviation of three biological replicates. No significant (n.s.) difference in cell growth is observed. Statistical testing: Mann Whitney test on the last time point.

D. Immunofluorescence microscopy of the indicated samples using the indicated antibodies, showing no detectable change in endogenous DNA damage, as assessed by staining for γH2A.X. Nuclei are counterstained with DAPI (blue). Scale Bar 10 µm.

E. Above, indel profiles at 96 target sites from van Overbeek *et al.* Below, normalized ChIP-seq signal for H3K9ac and H3K27ac and DNase-seq signal in a 500-nucleotide window centred on the cleavage site. Data presented from HEK293 (H) and K562 (K) cells (Cistrome DB 43073, 45020, 45021, 45406, 55731, 58997 and GEO GSM1635901 - 6).

F. Examples of two imprecise (left and middle) and one precise (right) sites showing highly different chromatin states in the indicated cell lines. For each target, tracks of the indicated histone marks and DNAse hypersensitivity sites (left) and the indel profiles (right) in the two cell lines are shown. The location of target sites is shown in blue below the tracks.

Figure S6. Related to Figure 6

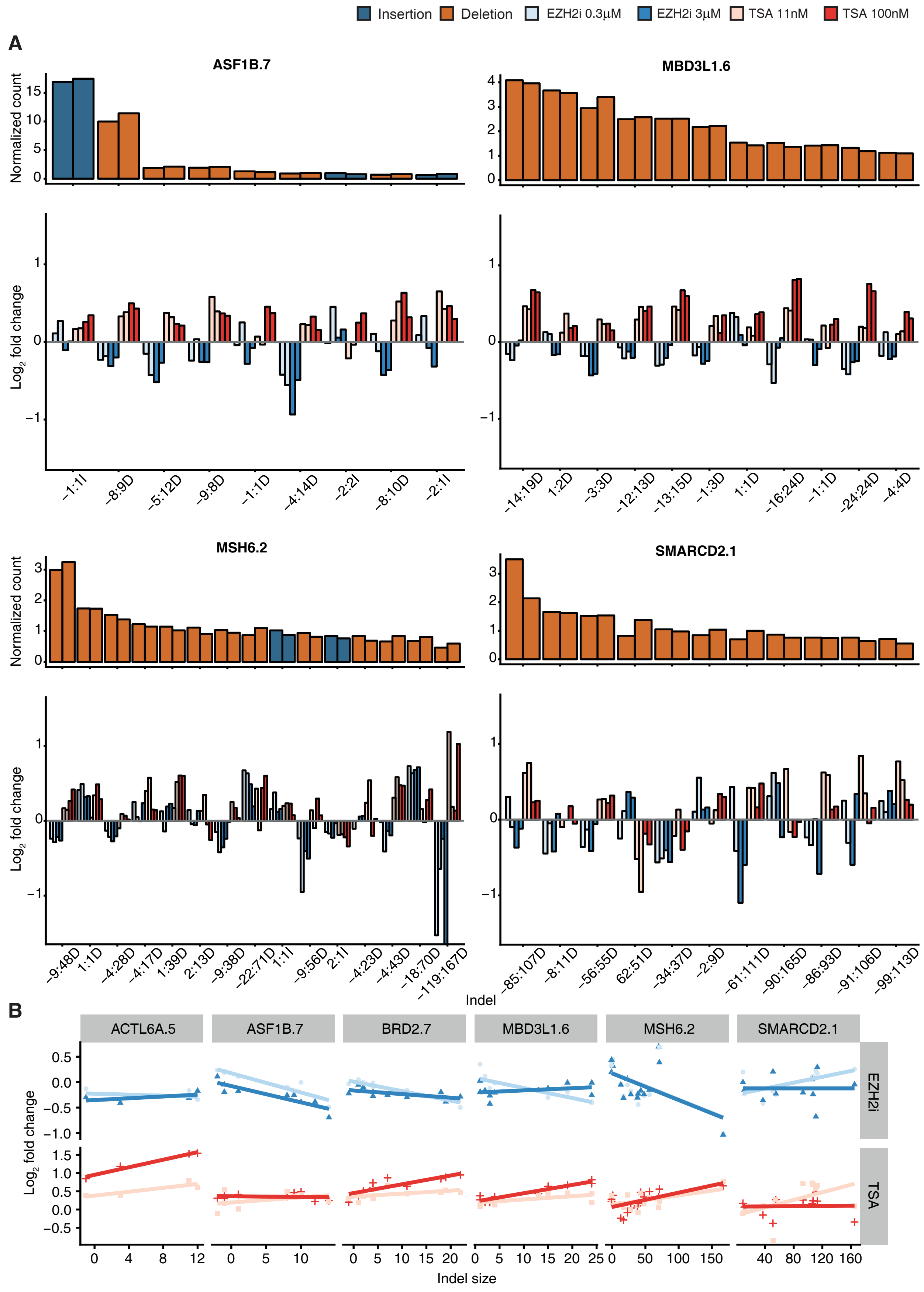

Figure S6. Chromatin modulation affects RGN activity. Related to Figure 6.

A. As for Figure 6D, the remaining 4 target sites show that chromatin modulation affects both insertions and deletions. Above is shown the count of each indel, normalized by the effective library size at each site for each replicate. Only indels with a normalized count of at least 1 in any condition are included.

B. Log_2-fold change for each size of indel for each target site and for each condition.

Figure S7. Related to Figure 7

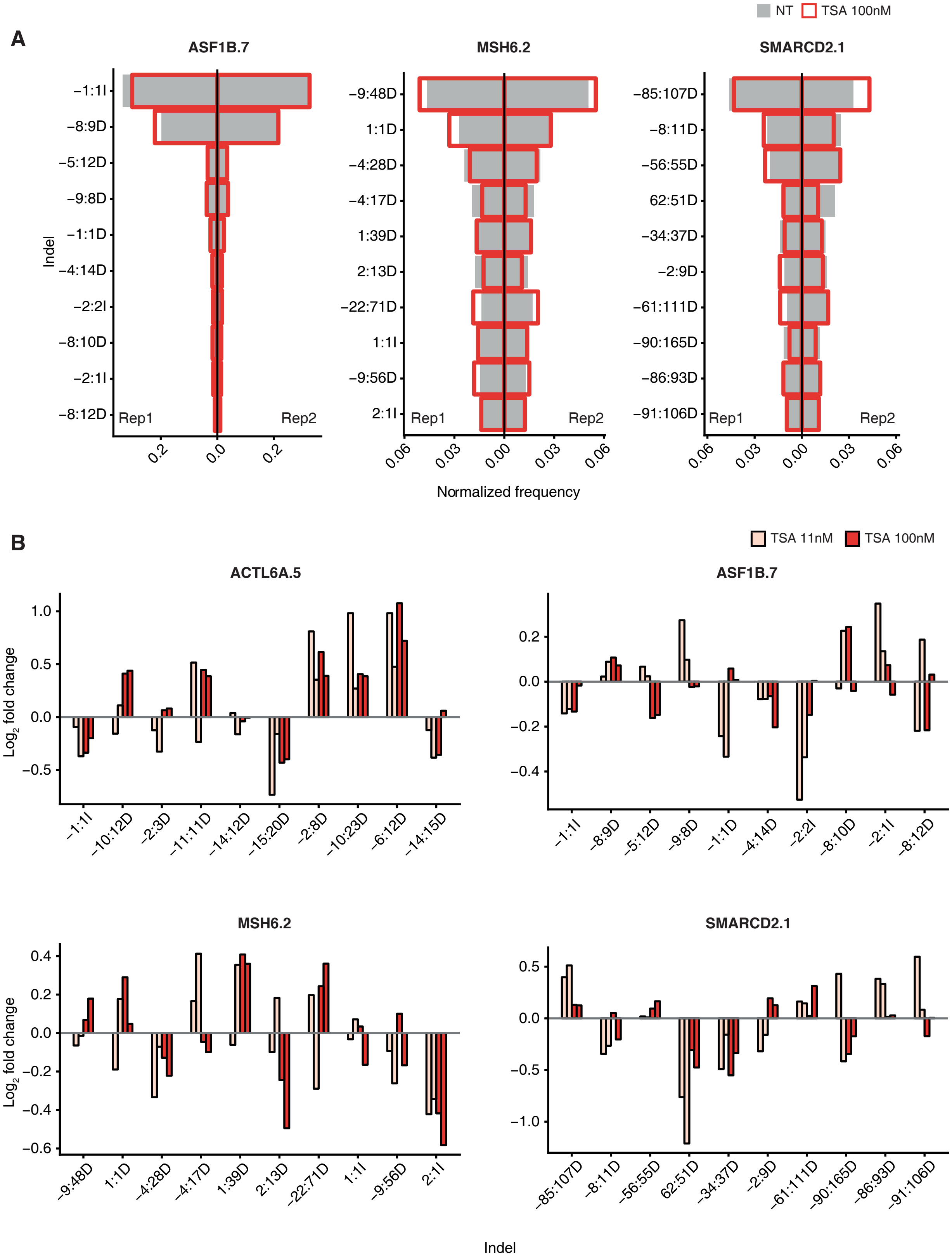

Figure S7. Chromatin modulation affects indel profiles. Related to Figure 7.

A. As for Fig. 7A, the remaining 3 targets show that chromatin modulation has a differential effect on distinct indels. Count for each indel, normalized by the total number of indels detected at that target site in that condition for each replicate. The frequency of the indicated indels in the untreated condition (grey bars) and in the TSA 100nM condition (red outline) is shown. The 10 commonest indels for each site are shown.

B. As for Fig. 7B, the $\log_2$ fold change in frequency for the indicated indels is shown for the remaining 4 target sites. The 10 commonest indels across both replicates are shown.

Table S5: Precision and Insertion rate associated with dinucleotides at -5 and -4 positions. Related to Figure 5
Dinucleotides represented by more than 10 sites are shown.

-5	-4	Median commonest frequency	Precise %	Middle %	Imprecise %	Insertion %	Deletion %	Number of sites
A	T	0.65	61.5	7.7	30.8	92.3	7.7	13
N	T	0.56	51.2	32.6	16.3	90.7	9.3	43
C	A	0.53	56.3	31	12.7	80.3	19.7	71
C	T	0.45	47.1	47.1	5.9	100		17
N	A	0.42	35	39.9	25.2	77.3	22.7	163
A	A	0.41	26	54	20	84	16	50
C	C	0.39	25	51.4	23.6	30.6	69.4	72
N	C	0.31	13.1	48.3	38.6	44.3	55.7	176
G	C	0.27	6.2	46.9	46.9	62.5	37.5	32
A	C	0.27	4.8	50	45.2	66.7	33.3	42
G	A	0.26	9.4	40.6	50	62.5	37.5	32
C	G	0.25	14.3	33.9	51.8	23.2	76.8	56
T	G	0.25	9.8	35.3	54.9	13.7	86.3	51
T	C	0.24	3.3	40	56.7	26.7	73.3	30
G	G	0.21	9.8	25.5	64.7	19.6	80.4	51
N	G	0.21	7.5	32.6	59.9	21	79	267
A	G	0.21	1.8	33.9	64.2	23.9	76.1	109
